# Molecular characterisation and genetic mapping of candidate genes for qualitative disease resistance in perennial ryegrass (*Lolium perenne *L.)

**DOI:** 10.1186/1471-2229-9-62

**Published:** 2009-05-19

**Authors:** Peter M Dracatos, Noel OI Cogan, Timothy I Sawbridge, Anthony R Gendall, Kevin F Smith, German C Spangenberg, John W Forster

**Affiliations:** 1 Department of Primary Industries, Biosciences Research Division, Victorian AgriBiosciences Centre, 1 Park Drive, La Trobe University Research and Development Park, Bundoora, Victoria 3083, Australia; 2 Department of Botany, Faculty of Science, Technology and Engineering, La Trobe University, Bundoora, Victoria 3086, Australia; 3 Department of Primary Industries, Biosciences Research Division, Hamilton Centre, Mount Napier Road, Hamilton, Victoria 3300, Australia; 4 Molecular Plant Breeding Cooperative Research Centre, Bundoora, Victoria, Australia

## Abstract

**Background:**

Qualitative pathogen resistance in both dicotyledenous and monocotyledonous plants has been attributed to the action of resistance (R) genes, including those encoding nucleotide binding site – leucine rich repeat (NBS-LRR) proteins and receptor-like kinase enzymes. This study describes the large-scale isolation and characterisation of candidate R genes from perennial ryegrass. The analysis was based on the availability of an expressed sequence tag (EST) resource and a functionally-integrated bioinformatics database.

**Results:**

Amplification of R gene sequences was performed using template EST data and information from orthologous candidate using a degenerate consensus PCR approach. A total of 102 unique partial R genes were cloned, sequenced and functionally annotated. Analysis of motif structure and R gene phylogeny demonstrated that *Lolium *R genes cluster with putative ortholoci, and evolved from common ancestral origins. Single nucleotide polymorphisms (SNPs) predicted through resequencing of amplicons from the parental genotypes of a genetic mapping family were validated, and 26 distinct R gene loci were assigned to multiple genetic maps. Clusters of largely non-related NBS-LRR genes were located at multiple distinct genomic locations and were commonly found in close proximity to previously mapped defence response (DR) genes. A comparative genomics analysis revealed the co-location of several candidate R genes with disease resistance quantitative trait loci (QTLs).

**Conclusion:**

This study is the most comprehensive analysis to date of qualitative disease resistance candidate genes in perennial ryegrass. SNPs identified within candidate genes provide a valuable resource for mapping in various ryegrass pair cross-derived populations and further germplasm analysis using association genetics. In parallel with the use of specific pathogen virulence races, such resources provide the means to identify gene-for-gene mechanisms for multiple host pathogen-interactions and ultimately to obtain durable field-based resistance.

## Background

Perennial ryegrass (*Lolium perenne *L.) is the most widely cultivated forage, turf and amenity grass species of global temperate grazing zones. Favourable agronomic qualities include high dry matter yield, nutritive content, digestibility, palatability and the ability to recover from heavy defoliation by herbivores [1,2]. Perennial ryegrass is, however, susceptible to a number of different foliar diseases. Crown rust (*Puccinia coronata *f.sp. *lolii*) is the most widespread and damaging disease affecting ryegrasses [3-7]. Stem rust (*P. graminis *f.sp. *lolii*) infections are especially serious for producers of ryegrass seed [8], while grey leaf spot (*Magnaporthe grisea*), dollar spot (*Sclerotinia homoeocarpa*) and brown patch (*Rhizoctonia solani*) reduce turf quality [9]. The development of cultivars resistant to each of these diseases is currently recognised as an important mode of infection control.

The obligate outbreeding reproductive habit of perennial ryegrass [10] leads to high levels of genetic variation within, and to a lesser extent, between cultivars [11-13]. Conventional breeding for disease resistance is hence anticipated to be relatively slow for outcrossing forage species as compared to allogamous species such as cereals, because of a requirement for extensive progeny screening and phenotyping. Nonetheless, major genes and quantitative trait loci (QTLs) for disease resistance have been detected in *Lolium *species for resistance to crown rust [14-21], stem rust [22], bacterial wilt [23], powdery mildew [24] and grey leaf spot [25]. The extent of genetic variation within temperate Australasian crown rust pathogen populations [26] is consistent with the presence of different virulence races [27]. Identification of the molecular basis of major resistance determinants to different pathotypes will improve selection of favourable alleles during cultivar development.

Both genetic and physiological analysis has determined that hypersensitive reactions in response to fungal, viral and bacterial pathogen infections are caused by the action of genes encoding receptor proteins [28,29]. The major class of resistance (R) genes contain a highly conserved nucleotide binding site (NBS) domain adjacent to the N-terminus and a leucine-rich repeat (LRR) domain involved in the host recognition of pathogen-derived elicitors. NBS-LRRs constitute one of the largest plant gene families, accounting for c. 1% of all open reading frames (ORFs) in both rice and *Arabidopsis thaliana*, and are distributed non-randomly throughout the genome [30-32]. Clustering of R genes is known to facilitate tandem duplication of paralogous sequences and generation of new resistance specificities to counter novel avirulence determinants in evolving pathogen populations [30-34].

NBS domain-containing sequences have been isolated using degenerate PCR from many agronomically-important Poaceae species including cereals [33-37] and forage grasses [24,38,39]. In a comparison with the fully-sequenced rice genome [31], only a small proportion of the total NBS domain sequences are so far likely to have been isolated from *Lolium *species. Multiple strategies are hence required to isolate a larger R gene sample, allowing for structural characterisation, marker development for genetic mapping, and the potential for correlation with the locations of known disease resistance loci.

Disease resistance loci of cereal species are conserved at the chromosomal and molecular level [40,41], and provide valuable template genes for a translational genomic approach to molecular marker development [42]. For example, the *Ta*Lrk10 receptor kinase gene (located at the *Lr10 *locus on hexaploid wheat chromosome 1AS) has been found to confer resistance to leaf rust in specific cultivars, and putative Lrk10 ortholoci are structurally conserved between Poaceae species [41,43]. The Lrk10 orthologue of cultivated oat (*Avena sativa *L.) exhibits 76% nucleotide similarity to the wheat gene and maps in a region of conserved synteny between the two genomes, co-locating with a large cluster of NBS-LRR genes conferring resistance to the oat form of crown rust (*P. coronata *f.sp. *avenae*) [41]. The Poaceae sub-family Pooideae includes perennial ryegrass, along with cereals of the Aveneae and Triticeae tribes [44,45], suggesting that template genes from these species are highly suitable for ortholocus isolation.

Based on studies of cereal-pathogen interactions, similar qualitative and quantitative genetic mechanisms are likely to contribute to disease resistance in perennial ryegrass. In order to test this hypothesis, a broad survey based on empirical and computational approaches was conducted to recover an enhanced proportion of perennial ryegrass NBS domain-containing sequences, as well as specific R gene ortholoci. Candidate R gene sequences (referred to as R genes throughout the text) were characterised by functional annotation, motif structure classification and phylogenetic analysis. Single nucleotide polymorphisms (SNPs) were discovered through re-sequencing of haplotypes from the parents of a two-way pseudo-testcross mapping population and validated SNPs were assigned to genetic maps. Co-location with disease resistance QTLs was demonstrated within *Lolium *taxa and by comparative analysis with related Poaceae species.

## Methods

### Bioinformatic approach to template gene selection

A proprietary resource of c. 50,000 perennial ryegrass expressed sequence tags (ESTs) [46] was integrated into the Bioinformatic Advanced Scientific Computing (BASC) system [47]. Each EST was functionally annotated using data from microarray-based transcriptomics experiments, the rice Ensembl browser, Pfam and gene ontology databases. BASC was used to search for the presence of NBS-LRR sequences. A text search with the query terms 'disease' and 'resistance' was used to identify candidates based on a wuBLASTX threshold of E = 10^-15 ^through known gene ontology within the genomes of closely-related cereal species (wheat, oat and barley), rice and *Arabidopsis*.

### Primer design for candidate Lolium R genes

Locus amplification primers (LAPs) for multiple target genes were designed using standard parameters as previously described [48]. LAPs were designed from perennial ryegrass EST templates, and sequence tagged site (STS) primers derived from Italian ryegrass (*L. multiflorum *Lam.) NBS sequences located in GenBank [39].

### Primer design based on Pooideae R gene templates

LAPs were designed based on the sequence of four oat LGB-located *Pca *cluster R genes [37], five barley rust resistance genes (*Hv*s-18, *Hv*s-133-2, *Hv*s-T65, *Hv*s-236 and *Hv*s-L6) [33]; and the third exon and 3'-terminus of the *Ta*Lrk10 extracellular domain [41].

### Degenerate primer design

Degenerate primers (4 in sense and 12 in antisense orientation) were designed to the conserved regions (P-loop and GLPL) of cloned oat R genes [37] and were used in conjunction with published R gene-specific degenerate primers [33,34,38,49] (Additional File 1). Based on interpretation of initial amplicon complexity, specific primers were subsequently designed for SNP discovery.

### Amplicon cloning and sequencing

For specific homologous and heterologous R gene-derived primers, PCR amplicons were generated using template genomic DNA from the parental genotypes of the F_1_(NA_6 _× AU_6_) mapping population [48,50]. For degenerate primers, genomic DNA from the crown rust resistant Vedette_6 _genotype [14] was used as an primary template, and re-designed primer pairs were used with the F_1_(NA_6 _× AU_6_) parents. Amplicons were cloned and sequenced essentially as previously described [48], except that a total of 32 Vedette_6 _clones and 12 clones from each of NA_6 _and AU_6 _were analysed. Trace sequence files were used as input materials into the BASC module ESTdB [47].

### Classification of derived sequences

All candidate NBS-LRR (R gene) nucleotide sequences were subjected to two-way BLASTX and wuBLASTX analysis against the GenBank and the Uniprot databases, respectively. Genomic DNA sequences were translated to amino acid sequences using Transeq software. Each peptide sequence was scanned against the Pfam database [51,52] for the presence of known domains, the type, size and position of NBS domains and the number of LRR repeats. Multiple Expectation Maximisation for Motif Elicitation (MEME) [53] was used to detect conserved motifs between sequences containing NBS domains [34].

### Phylogenetic analysis of R gene sequences

Preliminary alignments of predicted protein sequences was performed manually using Bioedit (version 7.0.5.3 – Ibis Biosciences, Carlsbad, CA, USA). The alignments were split into two separate datasets (for the P-Loop to GLPL region, and for the Kin-2A to GLPL region), and were realigned for phylogenetic analysis using CLUSTALX [54] with default options. Clustering of related sequences based on amino acid homology was conducted using a Neighbour Joining (NJ) algorithm and bootstrap analysis was calculated on an unrooted NJ cladogram based on 1000 iterations using CLUSTALX [55].

### Plant materials

Perennial ryegrass genomic DNA was extracted from parents and progeny of the F_1_(NA_6 _× AU_6_), Vedette_6 _and p150/112 [45,56] mapping families using the CTAB method [57]. A genotypic panel for genetic map assignment was constructed of 141 F_1_(NA_6 _× AU_6_) and 24 p150/112 F_1 _genotypes as previously described [21].

### *In vitro *discovery, validation and mapping of gene-associated SNPs

PCR-amplified genomic amplicons were cloned and sequenced and DNA sequences were aligned essentially as previously described [48]. Predicted SNPs were initially validated using 10 F_1_(NA_6 _× AU_6_) individuals, and those showing Mendelian segregation were then genotyped across the full mapping panel through the single nucleotide primer extension (SNuPe) assay [48]. Integration of SNP loci into the existing F_1_(NA_6 _× AU_6_) parental genetic maps was performed as previously described [21,48,50].

### Comparative genetic mapping

Comparison of chromosomal regions controlling crown rust resistance between perennial ryegrass trait-specific mapping populations was performed using data from QTL analysis of the F_1_(SB2 × TC1) mapping population [17]. The F_1_(SB2 × TC1) parental maps contained heterologous RFLP and genomic DNA-derived SSR (LPSSR) markers shared with the p150/112 and F_1_(NA_6 _× AU_6_) genetic maps, respectively [45,56]. Comparison of marker locus order between the p150/112 and F_1_(NA_6 _× AU_6_) genetic maps was performed through the presence of common LPSSR loci [50,56]. This common marker set also allowed interpolation of the position of the *LpPc*1 crown rust resistance locus on p150/112 LG2 [14]. Chromosomal locations of LrK10 ortholoci were compared between *Lolium *and *Avena *species using common heterologous RFLP loci [58]. Further comparative genomic analysis was conducted using published genetic maps from cereal species including barley, wheat, rye and oat [33,59].

## Results

### Strategies for specific R gene isolation

**Figure 1:**
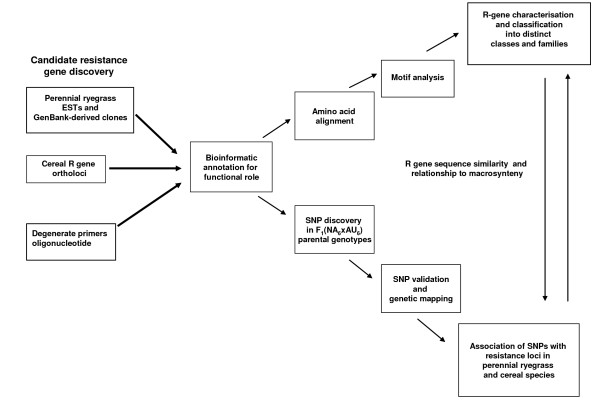
**Schematic representation of empirical and bioinformatics-based discovery of perennial ryegrass R genes**. Subsequent bioinformatic analysis leads to two streams of genetic analysis, including sequence characterisation, *in vitro *SNP discovery and large-scale genetic mapping.

Three strategies (empirical approaches based on heterologous PCR and degenerate PCR, and a bioinformatic discovery method) resulted in the identification of 67 primary R gene templates for host genetic analysis (Table 1). Initial PCR amplification and resequencing using the parental genotypes of the F_1_(NA_6 _× AU_6_) mapping population allowed identification of a further 35 secondary R gene template sequences (Additional File 2). A total of 14 primer pairs amplified paralogous sequences, at a mean of 2.5 per primary template sequence, with a range from 1–12. A total of 102 distinct putative R gene sequences (corresponding to 99 NBS-containing genes and 3 receptor kinase genes) were annotated (Additional File 2) and subjected to further characterisation. Representative genomic sequence haplotypes were deposited as accessions for unrestricted access in GenBank (accession numbers FI856066–FI856167). A schematic summary of the candidate gene discovery process and further applications is depicted in Figure 1.

**Table 1:** Classification of primary R gene templates used for host-specific genetic analysis, according to isolation strategy

**Perennial ryegrass unique identifier (UI)**	**Source of primary R gene template sequence**	**Reference**
***Primer design based on Pooideae R gene templates***		

*Lp*Lrk10	Wheat leaf rust receptor kinase	[41]

*Lp*PcaClone1	Oat NBS-LRR candidate from *Pca *cluster	[37]
*Lp*PcaClone2	Oat NBS-LRR candidate from *Pca *cluster	
*Lp*PcaClone3	Oat NBS-LRR candidate from *Pca *cluster	
*Lp*PcaClone4	Oat NBS-LRR candidate from *Pca *cluster	

*Lp*HvClone1	Barley NBS-LRR co-locating with QTL	[33]
*Lp*HvClone2	Barley NBS-LRR co-locating with QTL	
*Lp*HvClone3	Barley NBS-LRR co-locating with QTL	
*Lp*HvClone4	Barley NBS-LRR co-locating with QTL	
*Lp*HvClone5	Barley NBS-LRR co-locating with QTL	

***Degenerate primer pair design***		

*Lp*RGcontig1	Degenerate primer pair pair	Additional File 1
*Lp*RGcontig2	Degenerate primer pair	
*Lp*RGcontig3	Degenerate primer pair	
*Lp*RG1NBS	Degenerate primer pair	
*Lp*RG2NBS	Degenerate primer pair	
*Lp*RG3NBS	Degenerate primer pair	
*Lp*RG4NBS	Degenerate primer pair	
*Lp*RG5NBS	Degenerate primer pair	
*Lp*RG6NBS	Degenerate primer pair	
*Lp*RG7NBS	Degenerate primer pair	
*Lp*NBS-LRR1	Degenerate primer pair	
*Lp*NBS-LRR2	Degenerate primer pair	
*Lp*NBS-LRR3	Degenerate primer pair	
*Lp*NBS-LRR4	Degenerate primer pair	
*Lp*NBS-LRR5	Degenerate primer pair	
*Lp*NBS-LRR6	Degenerate primer pair	
*Lp*NBS-LRR7	Degenerate primer pair	
*Lp*NBS-LRR8	Degenerate primer pair	
*Lp*NBS-LRR9	Degenerate primer pair	
*Lp*NBSC1	Degenerate primer pair	
*Lp*NBSC2	Degenerate primer pair	
*Lp*NBSC5	Degenerate primer pair	
*Lp*NBSC8	Degenerate primer pair	
*Lp*NBSC15	Degenerate primer pair	
*Lp*DEGVed1_d03_gp08	Degenerate primer pairs designed to oat NBS	
*Lp*DEGVed2_d07_gp09	Degenerate primer pairs designed to oat NBS	
*Lp*DEGVed3_a11_gp09	Degenerate primer pairs designed to oat NBS	
*Lp*DEGVed4_d02_gp08	Degenerate primer pairs designed to oat NBS	

***Primer design for candidate *Lolium *R genes***		

*Lp*ESTa03_10rg	*Lp*EST from bioinformatic discovery	[46]
*Lp*ESTa08_14rg	*Lp*EST from bioinformatic discovery	
*Lp*ESTa10_13rg	*Lp*EST from bioinformatic discovery	
*Lp*ESTb02_05rg	*Lp*EST from bioinformatic discovery	
*Lp*ESTb06_11rg	*Lp*EST from bioinformatic discovery	
*Lp*ESTc10_19rg	*Lp*EST from bioinformatic discovery	
*Lp*ESTd08_13rg	*Lp*EST from bioinformatic discovery	
*Lp*ESTe01_10rg	*Lp*EST from bioinformatic discovery	
*Lp*ESTe11_14rg	*Lp*EST from bioinformatic discovery	
*Lp*ESTf06_19rg	*Lp*EST from bioinformatic discovery	
*Lp*ESTf11_11rg	*Lp*EST from bioinformatic discovery	
*Lp*ESTg01_20rg	*Lp*EST from bioinformatic discovery	
*Lp*ESTg04_17rg	*Lp*EST from bioinformatic discovery	
*Lp*ESTg06_13rg	*Lp*EST from bioinformatic discovery	
*Lp*ESTh04_17rg	*Lp*EST from bioinformatic discovery	
*Lp*ESTh05_28rg	*Lp*EST from bioinformatic discovery	
*Lp*ESTh07_17rg	*Lp*EST from bioinformatic discovery	
LPCL_38150	*Lp*EST from bioinformatic discovery	
LPCL_8913	*Lp*EST from bioinformatic discovery	
*Lp*HvESTClone1	*Lp*EST from bioinformatic discovery	
*Lp*HvESTClone2	*Lp*EST from bioinformatic discovery	
*Lp*HvESTClone3	*Lp*EST from bioinformatic discovery	
*Lp*HvESTClone4	*Lp*EST from bioinformatic discovery	

*Lp*AG205017	RG sequence from Italian ryegrass	[39]
*Lp*AG205018	RG sequence from Italian ryegrass	
*Lp*AG205035	RG sequence from Italian ryegrass	
*Lp*AG205050	RG sequence from Italian ryegrass	
*Lp*AG205055	RG sequence from Italian ryegrass	
*Lp*AG205063	RG sequence from Italian ryegrass	

*Lp*EST = perennial ryegrass EST; RG = resistance gene.

In the empirical approach category, translational genomics between perennial ryegrass and closely related cereal species (oat, barley and wheat) which are susceptible to other *Puccinia *rust pathogens (*P. coronata *f. sp. *avenae*, *P. hordii*, *P. triticina*) was used to identify R genes. Perennial ryegrass amplicons derived from oat R gene template primer pairs demonstrated high BLASTX similarity matches to their corresponding template sequences (data not shown). Primer pairs designed to the *Ta*Lrk10 template generated two 1.6 kb fragments, one of which (*Lp*Lrk10.1) displayed very high similarity scores to the putative oat ortholocus (*As*Pc68LrkA).

The specificity of amplification using degenerate primers designed to amplify NBS domains was dependent on the proportion of deoxyinosine (I)-containing nucleotides. Those based on oat R gene templates contained a high frequency of inosines (>15%) and predominantly amplified retrotransposon-like sequences (data not shown). In contrast, combinations of largely non-degenerate primer pairs based on sequence information from multiple Poaceae species (barley, sorghum and ryegrass) (Additional File 1), successfully generated NBS domain-containing amplicons of the correct size (Additional File 3). A total of 28 distinct NBS domain-containing sequences (Tables 1, Additional File 2) were generated, several primer pairs generating multiple products (up to 7) (Additional File 3).

The text search-based computational approach identified 23 distinct perennial ryegrass ESTs with high sequence similarity to known resistance genes from closely-related species (Table 1, Additional File 2). Amplification based on candidate EST primary templates was efficient, with only 13% of LAP pairs failing to generate amplicons. Additional sequences were amplified from several ESTs, all were putative paralogues showing significant BLASTX similarity (E < 1 × 10^-15^) to known R genes (Additional File 2).

Database searches for previously-characterised ryegrass NBS sequences identified 51 accessions from Italian ryegrass-derived clones and a further 14 from an interspecific *L. perenne *× *L. multiflorum *hybrid (*L. x. boucheanum*). All 6 previously-described STS primer pairs successfully generated single amplicons of the expected size (Table 1, Additional File 2).

### Molecular characterisation of perennial ryegrass R genes

From the total of 102 analysed sequences, 89 (87%) exhibited BLASTX matches at E < 10^-20 ^to known NBS domain-containing sequences from closely-related cereal species in both the GenBank and UniProt databases (Additional File 2). In most cases (80%), the highest matching sequence was the same for both databases. Sequence translation and subsequent Pfam analysis revealed that a substantial proportion of partial protein sequences were similar to the NBS domain (Additional File 4). A large proportion of the NBS-category sequences (55%) were within the NBS domain, while the remaining sequences either overlapped the NBS region at the N- or C- terminus, contained the LRR domain, or were located solely within the N- or C- terminal domain. A range of different R gene sub-classes containing NBS, CC-NBS, NBS-LRR, NBS-NBS-LRR, CC-NBS-LRR, CC-CC-NBS-LRR and NBS-NBS domains were detected, but no TIR-NBS containing sequences were observed. Of the different sub-classes of NBS sequences, 52 contained 1–33 LRRs (modal at 3), 25 contained 1 or more CC domains, and five sequences contained the NBS-NBS domain (Additional File 4). A further three receptor kinase and NBS-LRR genes contained trans-membrane domains.

Consensuses were determined for the seven major NBS domain motifs (P-Loop, RNBS-I, Kin-2A, RNBS-II, RNBS-III, GLPL and RNBS-V) (Additional File 5) and were compared to those from closely related Poaceae species (wheat and rice) and to *A. thaliana*. The P-Loop, Kin-2A and GLPL motifs were most highly conserved between all species examined, while the RNBS-I and RNBS-II motifs were conserved within the Poaceae, and the Kin-2A and RNBS-II motifs were the most conserved among the CC-NBS sequences. The RNBS-III and RNBS-V motifs were highly divergent between all species.

**Figure 2:**
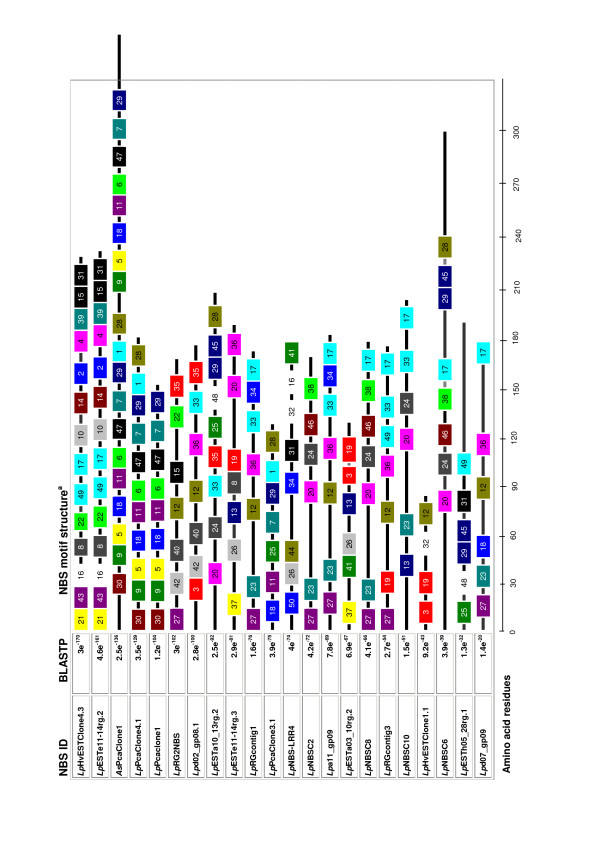
**Representation of motif patterns in the NBS domain of perennial ryegrass R gene sequences**. Different coloured boxes and numbers indicate distinct motifs identified by the MEME program which are displayed using the MAST application (details provided in Additional File 6).

A total of 50 different motif signatures were identified by MEME analysis with 60 NBS domain-containing sequences at an average of 13 residues in length. The most commonly occurring signatures were components of the conserved regions such as the P-Loop, Kin-2A and GLPL motifs (Fig. 2, Additional File 6). All the distinct sub-classes of NBS sequences present either completely lacked, or contained highly variable RNBS regions. Structural analysis revealed substantial diversity in motif content within the NBS domain and grouping of specific motifs into sub-classes based on shared sequence origin.

### Phylogenetic analysis of perennial ryegrass R genes

Phylogenetic analysis was performed based on two selected NBS domain regions (P-Loop-GLPL and Kin-2A-GLPL). Unrelated NBS domain sequences from *A. thaliana*, lettuce (*Lactuca sativa *L.), flax (*Linum usitatissimum *L.), tomato (*Lycopersicon esculentum *L.) oat, rice and barley were included for both regions, as were GenBank-derived *Lolium *NBS sequences. A total of 38 P-Loop-GLPL sequences and 104 Kin-2A-GLPL sequences were analysed. Amino acid alignment of NBS regions permitted classification into sub-families or classes. A total of seven major clusters were identified for the P-Loop-GLPL region (Additional File 7, Additional File 8). Candidate sequences were clustered on the basis of similarity to putative orthologues identified from preliminary BLASTX analysis. The majority were most closely related to those from other ryegrass species, although some showed highest sequence similarity to template genes from other species. Sequences similar to rice R genes were also grouped with flax, lettuce and *A. thaliana *accessions [cluster C], and a sub-set of ryegrass sequences formed two separate clusters [clusters G and H] and may hence be similar to generic R gene variants previously identified in other species, which were not included within the alignment.

Eight major clusters were identified for the Kin-2A-GLPL region (Additional File 9, Additional File 10). Ryegrass-derived sequences were preferentially clustered with those from other Poaceae species (for instance, with oat sequences formerly used as LAP-design templates [cluster A], and with rice and barley sequences [clusters C and G, respectively]). Sequences from a number of dicotyledonous plant species were separately clustered for the P-Loop-GLPL [cluster E], but co-located in several distinct clusters [cluster D and E] with ryegrass-derived sequences for the Kin-2A-GLPL region.

### *In vitro *SNP discovery and genetic mapping of perennial ryegrass R genes

Sixty-five distinct R gene templates were subjected to *in vitro *SNP discovery through resequencing from parental genotypes of the F_1_(NA_6 _× AU_6_) mapping population. Genomic DNA of a cumulative length of c. 37 kb was analysed and a total of 819 R gene SNPs were predicted, at an overall frequency of 1 per 46 bp. A total of 11 (17%) template biparental contigs contained no SNPs, while 27 (42%) of the remaining templates contained under 10 SNPs (Table 2). All monomorphic R gene contigs were derived from the NBS domain, apart from two encoding receptor kinase-like enzymes. SNP incidence was low within introns, due to limited representation in the sample set. SNP frequencies within parental genotypes was higher for NA_6 _(38) than for AU_6 _(20). A further 8 SNPs with biparental (AB × AB) segregation structures and 4 SNPs with AA × BB structures were identified.

**Table 2:** Summary information for *in vitro *SNP discovery and genetic mapping of candidate R gene SNPs

**Perennial ryegrass unique identifier (UI)**	**R gene SNP locus Identifier**	**Number of putative SNPs/contig size (bp)**	**SNP frequency (per bp)**	**Number of SNPs validated in panel of10 F_1_(NA_6 _× AU_6_) progeny**	**LG location and mapped locus coordinate (cM)** **[F_1_(NA_6 _× AU_6_)]**	**LG location and mapped locus coordinate (cM)** **[p150/112 population]**
*Lp*LrK10.1	rg1	15/1500	107	1	NA_6_-LG1- 34.7	N/A
*Lp*PcaClone 1.1	rg2	3/358	203	0	N/A	N/A
*Lp*PcaClone 1.2	rg3	0/470	N/A	0	N/A	N/A
*Lp*PCAClone2.1	rg4	0/380	N/A	0	N/A	N/A
*Lp*PcaClone3.1	rg5	1/510	510	0	N/A	N/A
*Lp*PcaClone3.2	rg6	0/187	N/A	0	N/A	N/A
*Lp*PcaClone3.3	rg7	0/187	N/A	0	N/A	N/A
*Lp*PcaClone4.1	rg8	1/510	510	1	NA_6 _– LG1- 151.6	N/A
*Lp*Hvclone1	rg9	0/250	N/A	0	N/A	N/A
*Lp*Hvclone2	rg10	0/230	N/A	0	N/A	N/A
*Lp*Hvclone3	rg11	2/406	N/A	0	N/A	N/A
*Lp*RGContig1	rg12	4/646	162	1	N/A	N/A
*Lp*RGContig2	rg13	2/504	86	1	AU_6 _– LG2- 57.4	LG2 – 32.5
*Lp*RGContig3	rg14	0/590	N/A	0	N/A	N/A
*Lp*RG1NBS	rg15	18/410	30	3	NA_6 _– LG2- 172.7	N/A
*Lp*RG2NBS	rg16	17/412	24	2	N/A	N/A
*Lp*RG3NBS	rg17	14/540	36	0	N/A	N/A
*Lp*RG4NBS	rg18	4/537	134	0	N/A	N/A
*Lp*RG5NBS	rg19	20/520	26	0	N/A	N/A
*Lp*RG6NBS	rg20	25/540	22	0	N/A	N/A
*Lp*RG7NBS	rg21	15/520	35	0	N/A	N/A
*Lp*NBSC5	rg22	3/423	141	1	N/A	N/A
*Lp*ESTa03_10rg.1	rg23	23/295	77	3	AU_6 _– LG1- 74.1	N/A
*Lp*ESTa08_14rg	rg24	24/723	15	2	NA_6 _– LG2- 166.6	LG2 – 62.9
*Lp*ESTa10_13rg	rg25	5/594	119	1	N/A	N/A
*Lp*ESTb06_11rg	rg26	10/729	45	3	AU_6 _– LG5 – 65.1	LG5 – 20.2
*Lp*ESTc10_19rg	rg27	9/550	96	2	NA_6 _– LG5 – 0.0; AU_6 _– LG5 – 68.4	LG5 – 19.2/19.7
*Lp*ESTd08_13rg	rg28	6/859	61	2	NA_6 _– LG5 – 10.9; AU_6 _– LG5 – 27.0	N/A
*Lp*ESTe11_14rg.1	rg29	14/684	49	1	NA_6 _– LG1 – 176.1	N/A
*Lp*ESTe11_14rg.2	rg30	14/684	49	2	AU_6 _– LG1 – 118.9	N/A
*Lp*ESTe11_14rg.3	rg31	12/591	66	2	NA_6 _– LG2 – 161.5	N/A
*Lp*ESTe11_14rg.4	rg32	16/605	38	1	N/A	N/A
*Lp*ESTe11_14rg.5	rg33	1/645	645	0	N/A	N/A
*Lp*ESTe11_14rg.7	rg34	0/375	N/A	0	N/A	N/A
*Lp*ESTe11_14rg.8	rg35	0/435	N/A	0	N/A	N/A
*Lp*ESTe11_14rg.9	rg36	3/423	141	0	N/A	N/A
*Lp*ESTe11_14rg.10	rg37	8/380	48	0	N/A	N/A
*Lp*ESTe11_14rg.11	rg38	0/690	N/A	0	N/A	N/A
*Lp*ESTe11_14rg 12	rg39	7/810	116	0	N/A	N/A
*Lp*ESTf06_19rg.1	rg40	9/550	61	3	NA_6 _– LG2 – 71.8/78.0	LG2 – 32.5
*Lp*ESTf11_11rg	rg41	35/890	25	0	N/A	N/A
*Lp*ESTg01_20rg	rg42	3/325	252	2	AU_6 _– LG3 – 45.9	N/A
*Lp*ESTg04_17rg.1	rg43	7/670	26	3	NA_6 _– LG4 – 92.8/94.9	N/A
*Lp*ESTg06_13rg	rg44	59/880	49	1	NA_6 _– LG2 – 164.4	N/A
*Lp*ESTg10_13rg.1	rg45	14/540	143	2	NA_6 _– LG5 – 37.1	N/A
*Lp*ESTg10_13rg.2	rg46	2/540	270	2	NA6 – LG7 – 0	N/A
*Lp*ESTh04_17rg	rg47	12/604	8	3	AU_6 _– LG6 – 137.7	N/A
*Lp*ESTh05_28rg.1	rg48	13/580	108	2	AU_6 _– LG5 -0.0	N/A
LPCL_8913	rg49	8/600	39	3	NA_6 _– LG6 – 134/134	N/A
*Lp*HvESTClone1.1	rg50	90/730	73	3	N/A	LG2 – 55.2
*Lp*HvESTClone1.2	rg51	2/550	225	0	NA_6 _– LG5 – 105.1/124.8	N/A
*Lp*HvESTClone1.3	rg52	6/556	93	0	N/A	N/A
*Lp*HvESTClone1.4	rg53	60/690	12	0	N/A	N/A
*Lp*HvESTClone2.1	rg54	26/670	66	3	NA_6 _– LG3 – 130.8	N/A
*Lp*HvESTClone3.1	rg55	98/1100	11	1	N/A	N/A
*Lp*HvESTClone4.1	rg56	3/646	215	1	N/A	N/A
*Lp*HvESTClone4.2	rg57	13/680	52	0	N/A	N/A
*Lp*HvESTClone4.3	rg58	0/270	N/A	0	N/A	N/A
*Lp*HvESTClone4.4	rg59	8/930	116	0	N/A	N/A
*Lp*AG205017	rg60	7/540	75	2	NA_6 _– LG7 – 46.5;, AU_6 _– LG7 – 45.9	N/A
*Lp*AG205018	rg61	7/601	13	2	AU_6 _– LG1 – 187.4	N/A
*Lp*AG205035	rg62	10/660	23	2	NA_6 _– LG3 – 37.8	N/A
*Lp*AG205050	rg63	14/610	44	1	N/A	N/A
*Lp*AG205055	rg64	10/664	86	3	NA_6 _– LG2 – 149.4; AU_6 _– LG2 – 86.9	N/A
*Lp*AG205063	rg65	7/602	86	1	NA_6 _– LG2 – 134.3	N/A

Information on SNP frequencies within F_1_(NA_6 _× AU_6_) biparental contigs, preliminary validation and positions on the parental maps of the F_1_(NA_6 _× AU_6_) and p150/112 population are provided as applicable. The key for conversion of nomenclature from R gene identifier to SNP locus identifier (rg notation) is also provided.

**Figure 3:**
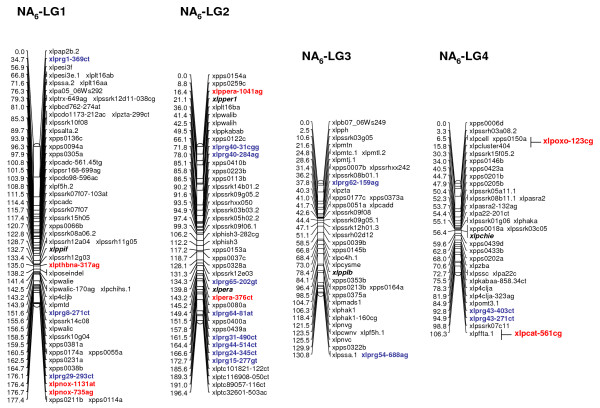
**Genetic linkage maps of LGs 1–4 from the NA_6 _parental genotype of the F_1_(NA_6 _× AU_6_) cross**. Nomenclature for the parental maps of the F_1_(NA_6 _× AU_6_) cross is as follows: EST-RFLP markers are indicated with xlp (co-dominant *Lolium perenne *locus) prefixes and gene-specific abbreviations, while EST-SSR are indicated with xpps prefixes, both as described in [50]; genomic DNA-derived (LPSSR) markers are indicated as xlpssr loci using the nomenclature described in [56]. SNP loci are designated according to the nomenclature xlp-gene name abbreviation-nucleotide coordinate-SNP identity [48]. For instance, xlpchijb-240cg on NA_6 _LG5 is derived from a chitinase class gene (*Lp*CHIjb), and the SNP is a C-G transversion located at coordinate 240. DR gene SNP loci are indicated in bold red type, and corresponding RFLP loci in black bold italic type. R gene SNP loci (designated with xlprg prefixes, and numbered according to Table 2), are indicated in bold blue type. Auxiliary DR and R gene loci mapped using JOINMAP 3.0, but not MAPMAKER 3.0, are interpolated between flanking markers to provide approximate genetic map locations.

**Figure 4:**
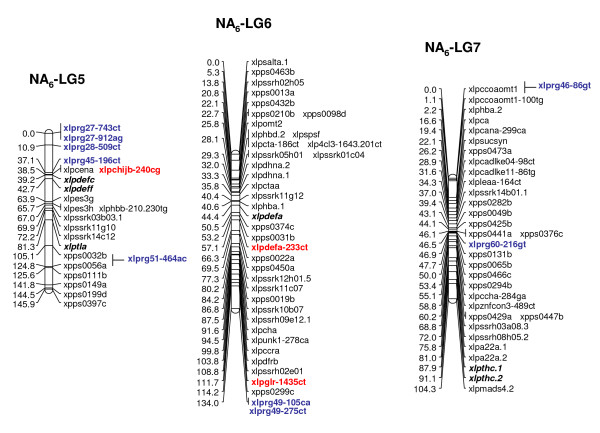
**Genetic linkage maps of LGs 5–7 from the NA_6 _parental genotype of the F_1_(NA_6 _× AU_6_) cross**. Details are as described in the legend to Fig. 3.

**Figure 5:**
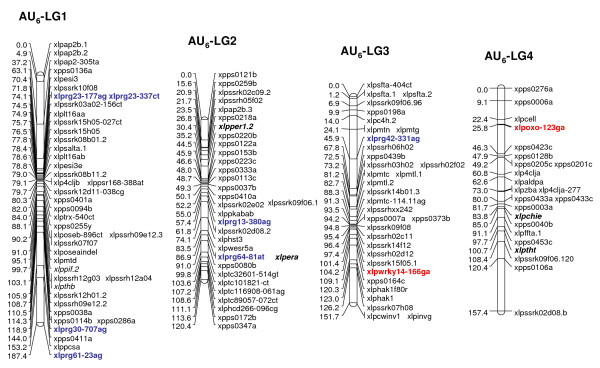
**Genetic linkage maps of LGs 1–4 from the AU_6 _parental genotype of the F_1_(NA_6 _× AU_6_) cross**. Details are as described in the legend to Fig. 3.

**Figure 6:**
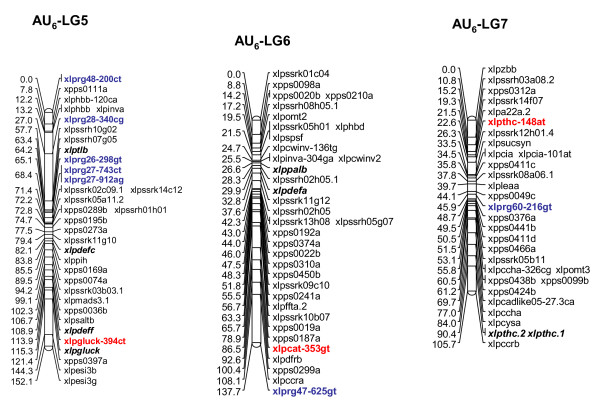
**Genetic linkage maps of LGs 5–7 from the AU_6 _parental genotype of the F_1_(NA_6 _× AU_6_) cross**. Details are as described in the legend to Fig. 3.

**Figure 7:**
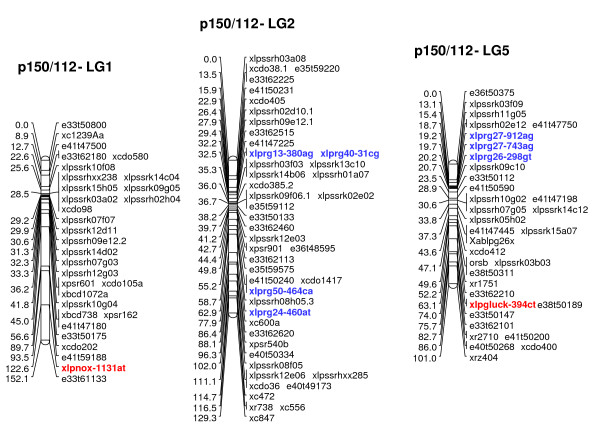
**Genetic linkage maps of LGs 1,2 and 5 from the p150/112 reference population**. Marker nomenclature for the p150/112 map is as follows: AFLP loci are indicated in the format exxtyyyyy (e.g., e33t50800) and heterologous RFLP loci are indicated as × plus the relevant probe name (e.g., xcdo580). Homologous RFLP loci detected by *Pst*I genomic clones are indicated as xablpgxxx (e.g.xablpg26y). Isoenzyme and EST markers are indicated with xlp prefixes and abbrevations for gene function (e.g. acp/2 and osw). Details of SNP loci are as described in the legend to Fig. 3.

Multiple R gene SNPs from 37 (69%) of 54 SNP-containing R gene contigs were validated (Additional File 11). A total of 26 R genes were assigned to loci on the parental maps of the F_1 _(NA_6 _× AU_6_) mapping population (22 on all NA_6 _LGs [Figs. 3, 4], 10 on all but LG4 for AU_6 _[Figs. 5, 6]). SNPs in four R gene loci showed biparental segregation structures, mapping to the equivalent LG position in each parental map, and hence provide bridging markers. Five loci were also mapped to equivalent positions on three p150/112 LGs. A single SNP locus derived from the template sequence *Lp*HvESTClone1.1 (xlprg50-464ca) was mapped in p150/112 but not in F_1_(NA_6 _× AU_6_) (Fig. 7)

R gene locus clusters were identified on a number of LGs, often in close proximity to mapped DR gene loci (represented by SNP and previously mapped EST-RFLP loci). Major clusters were identified in the lower regions of LGs 1 and 2 and the upper region of LG5 of both F_1 _(NA_6 _× AU_6_) parental maps (Fig. 3, 4, 5, 6).

### Comparative genetic mapping based on R gene loci

**Figure 8:**
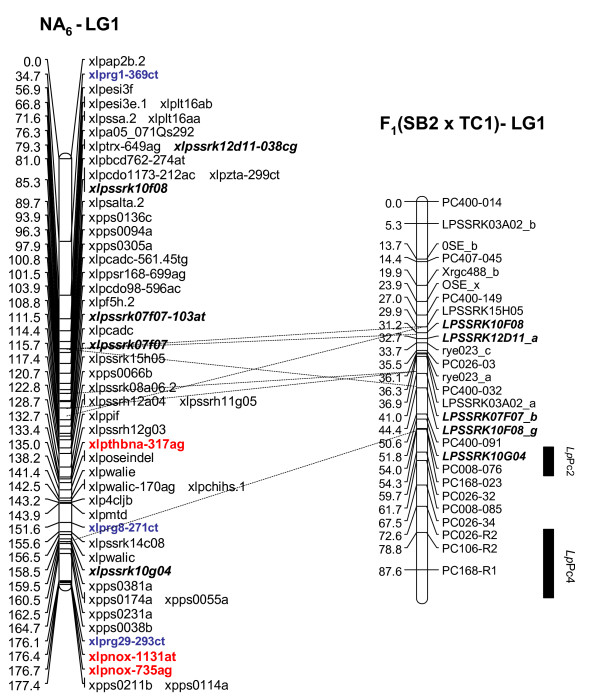
**Comparative mapping analysis between candidate R gene SNP loci mapped in the F_1_(NA_6 _× AU_6_) population and QTLs for crown rust resistance from other published studies**. Alignment of NA_6_-LG1 with *Lp*Pc2 and *Lp*Pc4 on LG1 F_1_(SB2 × TC1) [17]. Marker nomenclature for the NA_6 _and AU_6 _maps is as described in [48,50] and the legend for Fig. 3. Marker nomenclature within the F_1_(SB2 × TC1) mapping population is described in [17].

**Figure 9:**
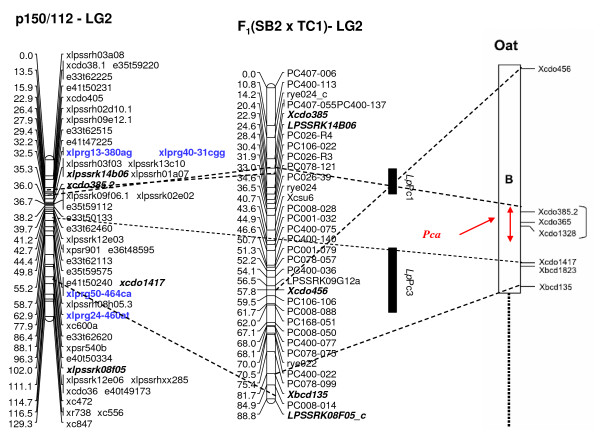
**Comparative mapping analysis between candidate R gene SNP loci mapped in the p150/112 population and QTLs for crown rust resistance from other published studies**. Alignment of p150/112-LG2 with the *Lp*Pc1 and *Lp*Pc3 loci on LG2 F_1_(SB2 × TC1) [17] and the *Pca *cluster on hexaploid oat LGB (adapted from [14]). Marker nomenclature for the p150/112 maps is as described in [48,50] and the legend for Fig. 7. The location of the *Lp*Pc1 crown rust resistance locus is as described in [14] and marker nomenclature within the F_1_(SB2 × TC1) mapping population is described in [17].

Genetic mapping facilitated map integration between trait-specific ryegrass genetic maps, and also comparative relationships with other *Lolium *and Poaceae taxa. Coincidences between SNP loci assigned to the F_1_(NA_6 _× AU_6_) parental maps and crown rust resistance QTLs detected in other studies were observed for LGs 1, 2, 5, and 7. Two R gene loci co-located with the crown rust resistance QTLs *Lp*Pc2 and *Lp*Pc4 in the lower region of LG1 (Fig. 8). A further two loci were assigned to the centromeric region of p150/112 LG2, 4 cM distant from the genomic DNA-derived SSR locus xlpssrk02e02 which is closely associated with *Lp*Pc1. This marker locus group also co-locates with *Lp*Pc3 in the F_1_(SB2 × TC1) LG2 map, and through comparative alignment, with the hexaploid oat *Pca *cluster on LGB based on the position of the heterologous RFLP locus xcdo385.2 (Fig. 9). The R gene SNP locus xlprg60-216gt mapped adjacent to a previously-identified crown rust resistance QTL on AU_6 _LG7, and in putative alignment with a corresponding QTL on LG7 of the *Lolium *interspecific hybrid ψ-F_2_(MFA × MFB) population map, but a limited number of common markers precluded further interpretation (data not shown).

**Figure 10:**
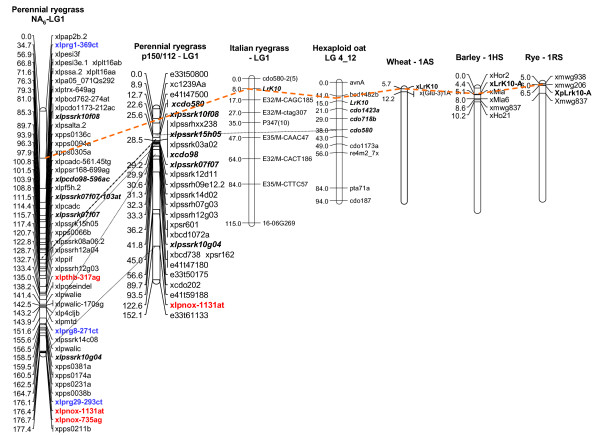
**Comparative mapping analysis of the perennial ryegrass LrK10 SNP locus (xlprg1-368ct)**. Macrosynteny of putative Lrk10 ortholoci was compared in other Poaceae species through alignment with LG1 of Italian ryegrass [76], LG4_12 from hexaploid oat, 1AS from wheat, 1HS from barley and 1RS from rye [41]. Black dotted lines align common genomic DNA-derived SSR markers (indicated in bold black italics) and an orange dotted line links the genetic map positions of LrK10 ortholoci.

Comparative genomic analysis detected conserved relationships between perennial ryegrass Lrk10 R gene SNP locus (xlprg1-369ct) and the corresponding cereal LrK10 template genes. A macrosyntenic region was identified on LG1, although low numbers of common genetic markers again limited the accuracy of extrapolation (Fig. 10). The perennial ryegrass R gene loci xlprg24-460at and xlprg54-688ag are derived from putative orthologues of the barley R genes HvS-217 and HvS-L8, respectively. Alignment of genetic maps revealed conserved syntenic locations, as well as coincidence with QTLs for leaf rust and powdery mildew resistance on barley 2H and 3H, respectively (Additional File 12, Additional File 13).

## Discussion

### Large-scale survey of perennial ryegrass NBS domain-containing sequences

This study describes the most comprehensive study to date of ryegrass NBS domain-containing sequences. The largest comparable surveys were of R genes from Italian ryegrass (62 sequences: [39]) and from both annual and perennial ryegrass and the corresponding interspecific hybrid (16 sequences: [38], all derived by means of degenerate primer-based amplification. In this study, 102 distinct R genes were isolated and functionally annotated. Bioinformatic analysis identified the majority of candidate genes as members of the NBS-LRR family responsible for major gene resistance in plant species [29,60-64]. A proportion of c. 20% of all perennial ryegrass R genes may be estimated to have been sampled, assuming equivalent gene content to that revealed (545 NBS sequences) by the genome-wide survey of rice [31]. It is possible, however, that major rounds of genome duplication or divergence events between species may have occurred, based on different selection pressures of surrounding pathogen populations. Such factors may influence the relative number of NBS-containing sequences in ryegrass species.

### Structural classification of perennial ryegrass NBS sequences

Results from the current study suggest that only non-TIR NBS sequences are present within the *Lolium *genome, consistent with previous results from monocotyledonous species [33,37-39,58,65]. Only degenerate primers specific to non-TIR sequences were able to amplify PCR products from perennial ryegrass genomic DNA, as observed in similar studies of sorghum [34].

Substantial variation was observed within coding regions of non-TIR NBS-LRRs, which exhibit greater sequence diversity than the TIR-NBS sub-family [66]. In this study, many R genes lacked the P-Loop region, while others contained NBS-NBS domains, duplicated CC regions or lacked CC and/or LRR domains. P-Loop, Kin-2A and GLPL motifs were conserved and similar in sequence to those of closely related Poaceae species such as wheat and rice [31,58] and the model dicotyledonous species *A. thaliana *[30]. Further evidence for structural gene diversity was observed within particular NBS sub-families. NBS sub-classes contained specific signature motifs between conserved regions, and in some instances, RNBS motifs were missing or duplicated. This suggests that the RNBS-I and RNBS-II motifs may either play a role in pathogen-specific recognition, or be less functionally significant than other, more highly conserved domains mediating resistance in plant species [30,66]. Alternatively, the presence of CC-NBS-specific motifs may suggest divergence to perform specialised functions. Variability was also observed within LRR domains, suggesting that NBS-LRRs in ryegrass are diverse in function [64,67].

### Phylogenetics of Lolium NBS domain-containing sequences and relationship to genomic location and evolution

Amino acid diversity in the P-Loop-Kin-2A region may account for the major differences between TIR-NBS and CC-NBS domains. The results from this study demonstrate that TIR-NBS sequences from flax and *A. thaliana *group in a separate cluster, as observed in a previous phylogenetic analysis of *Lolium *NBS domains [38]. Further sequence analysis of a larger number of *Lolium *sequences in the Kin-2A-GLPL motif interval demonstrated increased sequence similarity with known TIR-NBS regions from dicotyledonous plant species, suggesting that this region may be more conserved across taxa. Consensus motif order and sequence composition indicates that the *Lolium *RNBS-I region may have diverged from that of dicotyledonous plants. Similar results were observed in other Poaceae species such as sorghum, for which RNBS-I consensus sequences showed significantly higher similarity to those of rice than to those of *A. thaliana *TIR-NBS genes [34].

Phylogenetic analysis of the P-Loop-GLPL and the Kin-2A-GLPL domains detected at least 8 NBS sub-classes, as compared to 5 separate clusters identified in a previous study [38]. Analysis of the larger number of Kin-2A-GLPL interval sequences obtained only one more cluster than for the P-Loop-GLPL interval, indicative of domain conservation. Inclusion of NBS sequences from other closely, and more distantly related, species permitted grouping of R genes and inference of possible common origins for R gene sub-families. Sequences amplified from oat templates clustered together with ryegrass template-derived R genes, suggestive of a common origin. Based on known mechanisms of R gene evolution, gene duplication and divergence prior to speciation within the Pooideae sub-tribe is likely to account for the sequence similarity between ryegrass and oat genes, corresponding to putative orthologues [49,61,68].

### Candidate R gene SNP discovery and genetic mapping

The SNP frequency observed within this study was marginally lower than that detected within a sub-set of 11 perennial ryegrass R genes across 20 diverse genotypes [69], but similar to that observed within DR genes [21] and a broad range of functionally-annotated candidate genes [48] in the F_1_(NA_6 _× AU_6_) mapping population. Eight R gene templates contained up to 90 SNPs per contig, possibly due to paralogous sequence alignment. Large numbers of haplotypes have been reported for other perennial ryegrass NBS-LRR genes, especially within variable LRR regions [69]. The data from this study suggests that allelic diversity within NBS domain is low compared to the highly variable LRR domain.

Previous studies identified significantly non-random chromosomal distributions of NBS-containing sequences [30,31]: 44 gene clusters were detected in the *japonica *sub-species of rice. Five major clusters containing two or more closely linked NBS-LRR genes, which frequently showed low mutual sequence similarity, were identified from only a small sub-set (26) of mapped perennial ryegrass R genes. This suggests that the gene location pattern in perennial ryegrass may be similar to that observed in other plant species. Unrelated R genes also mapped in close association with DR gene SNP and RFLP loci [21,50]. QTL based analysis and genetic mapping in wheat identified co-location of DR and R genes at qualitative disease resistance loci [70,71]. Co-location of R genes with DR genes was also observed in similar chromosomal regions (lower regions of LG1, LG2 and LG6) as disease resistance QTLs which were mapped both in F_1_(NA_6 _× AU_6_) and other trait-specific mapping populations [17,20,21].

### Co-location of R gene SNP markers with disease resistance QTLs

SNP mapping of two candidate R genes in both the F_1_(NA_6 _× AU_6_) and p150/112 mapping populations has provided possible candidates for the major gene crown rust resistance QTL (*Lp*Pc1) on LG2 [14]. To determine whether R gene SNP variants are of functional significance, further experiments involving transgenic approaches, association genetic analysis or map-based cloning are required [72,73].

NBS-LRR genes loci mapping to the distal region of LG1 in the F_1_(NA_6_x AU_6_) parental genetic maps (xlprg29-293at, xlprg30-707ag and xlprg61-23ga) are potential candidates for resistance effects to crown rust pathotypes which are yet to be identified within Australasia. Major QTLs for crown rust resistance (*Lp*Pc2 and *Lp*Pc4) have been mapped to the lower part of LG1 in 3 different perennial ryegrass trait-specific mapping populations [17-19] but the limited number of common markers limits accurate extrapolation between genetic maps. Two QTLs of large magnitude were identified in each of the three populations, which were screened using European crown rust isolates However, so far no resistance QTLs have been detected within this chromosomal region using isolates from the southern hemisphere. As both F_1_(NA_6 _× AU_6_) mapping population parental genotypes are derived from Eurasia [50], LG1-located R gene polymorphisms may confer resistance to crown rust isolates of European provenance.

### Comparative genomics analysis of perennial ryegrass R genes

Comparative analysis of R gene SNP loci and corresponding ortholoci confirmed previously reported macrosyntenic relationships between perennial ryegrass and other Poaceae species [45] in nearly all instances. The sole exception was the xlprg8-271ct locus, which was assigned to LG1 despite being derived from (and highly similar to) an oat *Pca *template gene predicted to map to LG2. Genetic mapping of the *Lp*Lrk10 locus to LG1 suggested that the structure and chromosomal location of this gene are highly conserved throughout the Pooideae [41,43,58]. The equivalent analysis for barley R gene ortholoci provides the basis for testing R gene functionality in response to a broader range of plant diseases, requiring significant improvements of pathogen phenotyping [74] and corresponding genetic analysis [42,75].

## Conclusion

This study has demonstrated that multiple approaches to R gene discovery, including the use of homologous and heterologous templates, can generate significant numbers of candidate genes for major disease resistance loci. An enhanced resource of R gene templates from perennial ryegrass has permitted evaluation of gene structural diversity and putative evolutionary origins. Efficient *in vitro *discovery methods allowed assignment of R gene-derived SNPs to genomic locations, revealing coincidence with pathogen resistance QTLs in ryegrasses, as well as comparative relationships with other grass and cereal species. R gene-associated markers are suitable for further evaluation and implementation in forage grass improvement programs.

## Authors' contributions

PD carried out the experimental work and the majority of analysis, prepared the tables and figures and the primary drafts of the manuscript, and contributed to finalisation of the text and journal-specific formatting. NC co-conceptualised the project and contributed to data analysis and text preparation. TS provided EST sequence information and assisted preparation of files for GenBank submission. TG co-conceptualised the project and contributed to text preparation. KS co-conceptualised the project and developed and contributed genetically-defined plant materials. GS provided EST sequence information and valuable editorial advice. JF co-conceptualised the project, provided overall project leadership, and co-developed interim and final drafts of the manuscript.

## Supplementary Material

Additional File 1 **Degenerate oligonucleotide primers used for NBS domain-containing sequence amplification**. Sequence information for primer synthesis was obtained from published data specific to barley, sorghum and perennial ryegrass. Click here for file

Additional File 2 **Bioinformatic (BLASTX and wuBLASTX) annotation of cloned and sequenced primary and secondary perennial ryegrass R gene templates to both GenBank and UniProt databases within the Bioinformatic Advanced Scientific Computing (BASC) database (as of June 2007 release)**. BASC is linked to the rice Ensemble Browser and Uniprot databases and employs known gene ontology and Pfam domain analysis to assign putative function to candidate sequences. Nomenclature of paralogous sequences is based on the unique identifier for the primary template sequence (e.g., *Lp*ESTe11_14) followed by a numerical suffix (.1, .2 etc.), e.g. *Lp*ESTe11_14rg.1. Click here for file

Additional File 3 **Summary details for specific R gene-directed degenerate primer pair combinations, as described in Additional File **1**, along with primer pair code, numbers of amplification products and corresponding R gene templates.** Click here for file

Additional File 4 **Functional characterisation of predicted translation products from perennial ryegrass candidate R genes**. All information was derived from Pfam links within the best-available Uniprot wuBLASTX hits in BASC. Pfam information was used to obtain the probable location of candidate sequences, protein size, position of NBS sequence and number of LRR repeats. Click here for file

Additional File 5 **Major protein sequence motifs in predicted *Lolium *NBS domains**. ^a^Motifs listed in the order of occurrence in the NBS domain of putative perennial ryegrass R genes. Perennial ryegrass motifs were named in accordance with descriptions obtained from both rice and *A. thaliana *[30,31,66]; ^b^Bioinformatic analysis using Pfam on putative R gene sequences identified all to be CC-NBS types (CNL denotes CC-NBS-LRR and TNL denotes TIR-NBS-LRR); ^c^Consensus amino acid sequences for *Lolium *NBS sequences were derived from MEME, while those for wheat were derived from [58]. Click here for file

Additional File 6 **Summary information of amino acid structure for NBS domain protein sequence motifs numbered in Fig. **2**, based on matching using the MEME program.** Click here for file

Additional File 7 **Reference information for sequences corresponding to individual clusters identified during phylogenetic analysis for the complete NBS domain (P-Loop-GLPL), as depicted in Additional File **8. Click here for file

Additional File 8 **NJ dendrograms based on amino acid alignment of the full-length (P-Loop – GLPL) regions of NBS protein domains encoded by *Lolium *R genes**. Bootstrap values are displayed as percentages of 1000 neighbour joining bootstrap replications. Bootstrap values at or greater than 65% are shown. Bars at the right of the dendrograms represent R gene sub-classes. Click here for file

Additional File 9 **Reference information for sequences corresponding to individual clusters identified during phylogenetic analysis for the Kin-2A-GLPL region of the NBS domain, as depicted in Additional File **10. Click here for file

Additional File 10 **NJ dendrograms based on amino acid alignment of the partial (Kin-2A -GLPL) regions of NBS protein domains encoded by *Lolium *R genes**. Details are as described in the legend for Additional File 8. Click here for file

Additional File 11 **Summary information for LAP and SNuPe primers used for predicted R gene SNP validation**. Information on segregation structure, parental polymorphism, SNP variant and successful genetic map assignment is included. All LAP PCRs and SNuPe reactions were designed for operating annealing temperatures of 55°C and 50°C, respectively. Click here for file

Additional File 12 **Comparative chromosomal positions of predicted putative orthologous R genes between perennial ryegrass and barley: *Lp*s-217 (coded as xlprg50-464ca) on p150/112 LG2 compared to *Hv*s-217 at the bottom of chromosome 2H. qLr represents a QTL for barley leaf rust resistance.** Click here for file

Additional File 13 **Comparative chromosomal positions of predicted putative orthologous R genes between perennial ryegrass and barley: *Lp*s-L8 (coded as xlprg54-688ag) on NA_6_-LG3 compared to *Hv*s-L8 at the bottom of chromosome 3H. qMIL represents a major QTL for powdery mildew resistance in barley.** Click here for file
