# Supplementary material for: Molecular characterisation and genetic mapping of candidate genes for qualitative disease resistance in perennial ryegrass (Lolium perenne L.)
Source: BMC Plant Biol. 2009 May 19;9:62. doi: 10.1186/1471-2229-9-62 (PMC2694799; doi:10.1186/1471-2229-9-62)
Supplement: Additional File 1 — Degenerate oligonucleotide primers used for NBS domain-containing sequence amplification. Sequence information for primer synthesis was obtained from published data specific to barley, sorghum and perennial ryegrass. [file 1471-2229-9-62-S1.doc]

**Additional File 1**

| **Primer Identifier** | **Sequence (5’-3’)** | **Motif** | **Source species** | **Reference** |
| --- | --- | --- | --- | --- |
| DEGBNHvF1  DEGBNHvR1  DEGGLHvR2  DEGH2027SbR  DEGH1146SbR  DEGH2019SbF  DEGH2018SbF  DEGH2017SbF  DEGLi*etal*. (2006)R2  DEGLi*etal*. (2006)lR1  DEGLi*etal*. (2006)R3  DEGLi*etal*. (2006)F1  PUBDegs1  PUBDegs2  PUBDegas1  PUBDegas2  PUBDegas3 | GGAATGGGNGGNGTNGGNAARAC  YCTAGTTGTRAYDATDAYYYTRC  ARIGCIARIGGIARICC  YCTIGGRAAIARIGCRCARTA  GGIGGIATIGGIAAAACIAC  GGIGGIWSIGGIAARACIAC  GCIGCIARIGGIARICC  AIISHIARIGGIARICC  CAGGGCAAGGGTAGACC  GGIGGIINTIGGIAARACIAC  GGIGGIYTIGGIAARACIAC  IARIGYIARIGGIARICC  GGTGGGGTTGGGAAGACAACG  GGIGGIGTIGGIAAIACIAC  CAACGCTAGTGGCAATCC  IAAIGCIAGIGGIAAICC  IAGAGCIAGIGGIAGICC | P-loop  GLPL  GLPL  GLPL  GLPL  P-Loop  P-Loop  P-Loop  GLPL  GLPL  GLPL  P-Loop  P-Loop  P-Loop  GLPL  GLPL  GLPL | barley  barley  barley  sorghum  sorghum  sorghum  sorghum  sorghum  perennial ryegrass  perennial ryegrass  perennial ryegrass  perennial ryegrass  N/A  N/A  N/A  N/A  N/A | [33]  [33]  [33]  [34]  [34]  [34]  [34]  [34]  [38]  [38]  [38]  [38]  [49]  [49]  [49]  [49]  [49] |
