# Supplementary material for: Molecular characterisation and genetic mapping of candidate genes for qualitative disease resistance in perennial ryegrass (Lolium perenne L.)
Source: BMC Plant Biol. 2009 May 19;9:62. doi: 10.1186/1471-2229-9-62 (PMC2694799; doi:10.1186/1471-2229-9-62)
Supplement: Additional File 2 — Bioinformatic (BLASTX and wuBLASTX) annotation of cloned and sequenced primary and secondary perennial ryegrass R gene templates to both GenBank and UniProt databases within the Bioinformatic Advanced Scientific Computing (BASC) database (as of June 2007 release). BASC is linked to the rice Ensemble Browser and Uniprot databases and employs known gene ontology and Pfam domain analysis to assign putative function to candidate sequences. Nomenclature of paralogous sequences is based on the unique identifier for the primary template sequence (e.g., LpESTe11_14) followed by a numerical suffix (.1, .2 etc.), e.g. LpESTe11_14rg.1. [file 1471-2229-9-62-S2.doc]

**Additional File 2**

| **Perennial ryegrass unique identifier (UI)** | **NCBI Accession number** | **GenBank Annotation information (BLASTX)** | **E value (BLASTX)** | **BASC wuBLASTX annotation** | **E value**  **(wuBLASTX)** |
| --- | --- | --- | --- | --- | --- |
| *Lp*Lrk10.1 | AY038002 | Receptor Kinase LRK10 (*Avena sativa*) | 1 x 10^-106^ | **Receptor kinase LRK14 *(Avena sativa)*** | 1.1 x 10^-45^ |
| *Lp*Lrk10.2 | NP_001060426 | kinase calmodulin (*Oryza sativa*) | 2 x 10^-20^ | Protein Kinase Calmodulin (*Oryza sativa*) | 1.1 x 10 ^-13^ |
| *Lp*PcaClone1.1 | CAD26856 | putative resistance (*Avena strigosa*) | 2 x 10^-64^ | NBS_LRR 1 (*Avena strigosa*) | 1.2 x 10 ^-54^ |
| *Lp*PcaClone1.2 | AAX84523 | powdery mildew PM (*Triticum aestivum*) | 1 x 10^-23^ | NBS_LRR 3(*Avena strigosa*) | 7.1 x 10 ^-19^ |
| *Lp*PcaClone2.1 | AAX84523 | powdery mildew PM (*Triticum aestivum*) | 3 x 10^-75^ | NBS_LRR 3(*Avena strigosa*) | 1.9 x 10 ^-40^ |
| *Lp*PcaClone2.2 | CAF03709 | NBS-LRR- 3 (*Avena strigosa*) | 4 x 10^-49^ | NBS_LRR 3(*Avena strigosa*) | 2.2 x 10 ^-62^ |
| *Lp*PcaClone3.1 | AAB96985 | NBS-LRR (*Oryza sativa*) | 5 x 10^-67^ | Putative R-gene (*Avena strigosa*) | 1.1 x 10 ^-44^ |
| *Lp*PcaClone3.2 | AAX84523 | powdery mildew PM (*Triticum aestivum*) | 1 x 10^-48^ | Putative R-gene (*Avena strigosa*) | 1.4 x 10 ^-40^ |
| *Lp*PcaClone3.3 | AAX84523 | powdery mildew PM (*Triticum aestivum*) | 1 x 10^-43^ | Putative R-gene (*Avena strigosa*) | 2.6 x 10 ^-34^ |
| *Lp*PcaClone4.1 | CAF03709 | NBS-LRR 3 (*Avena strigosa*) | 5 x 10^-67^ | Putative R-gene (*Avena strigosa*) | 2.2 x 10 ^-61^ |
| *Lp*PcaClone4.2 | AAX84523 | powdery mildew PM (*Triticum aestivum*) | 7 x 10^-45^ | Putative R-gene (*Avena strigosa*) | 7.6 x 10 ^-68^ |
| *Lp*PcaClone4.3 | CAD26856 | putative resistance (*Avena strigosa*) | 2 x 10^-52^ | NBS_LRR 3 (*Avena strigosa*) | 3.9 x 10 ^-52^ |
| *Lp*HvClone1 | AAZ93563 | BN1-D5 (*Hordeum vulgare*) | 2 x 10^-05^ | NBS-LRR S-120 (*Hordeum vulgare)* | 1.6 x 10 ^-36^ |
| *Lp*HvClone2 | CAD44585 | NBS-LRR (*Hordeum vulgare*) | 8 x 10^-39^ | RGA S-215 NBS-LRR (*Hordeum vulgare)* | 1.4 x 10 ^-28^ |
| *Lp*HvClone3 | CAD44585 | NBS-LRR (*Hordeum vulgare*) | 1 x 10^-29^ | RGA S-215 NBS-LRR (*Hordeum vulgare)* | 2.3 x 10 ^-26^ |
| *Lp*HvClone4 | CAD44585 | NBS-LRR (*Hordeum vulgare*) | 3 x 10^-27^ | RGA S-215 NBS-LRR (*Hordeum vulgare)* | 1.3 x 10 ^-30^ |
| *Lp*HvClone5 | CAD44585 | NBS-LRR *(Hordeum vulgare*) | 7 x 10^-32^ | RGA S-215 NBS-LRR (*Hordeum vulgare)* | 7.2 x 10 ^-30^ |
| *Lp*RGcontig1 | AAX19072 | NBS resistance protein (*Lolium perenne*) | 2 x 10^-72^ | Resistance (*Thinopirum intermedium*) | 1.1 x 10^-62^ |
| *Lp*RGcontig2 | BAC15497 | putative resistance RPH8A (*Oryza sativa*) | 2 x 10^-54^ | Putative disease RPH8A (*Oryza sativa*) | 1.5 x 10^-34^ |
| *Lp*RGcontig3 | CAD45033 | NBS-LRR (*Hordeum vulgare*) | 4 x 10^-76^ | NBS-LRR disease (*Oryza sativa*) | 1.2 x 10^-69^ |
| *Lp*RG1NBS | AAX19079 | NBS (*Lolium perenne*) | 2 x 10^-93^ | NBS-LRR like Rice (*Oryza sativa*) | 5.3 x 10^-66^ |
| *Lp*RG2NBS | AAX19077 | NBS (*Lolium perenne*) | 2 x 10^-88^ | NBS-LRR like Rice (*Oryza sativa*) | 6.6 x 10^-68^ |
| *Lp*RG3NBS | ABA96074 | NB_ARC (*Oryza sativa*) | 2 x 10 ^-12^ | RHGB2 (*Hordeum vulgare*) | 0.26 |
| *Lp*RG4NBS | NP_001046563 | NB ARC (*Oryza sativa*) | 6 x 10^-64^ | Resistance I2 (*Oryza sativa*) | 1.7 x 10 ^-34^ |
| *Lp*RG5NBS | NP_001066382 | NB ARC (*Oryza sativa*) | 3 x 10^-38^ | Putative resistance (*Oryza sativa*) | 7.8 x 10 ^-11^ |
| *Lp*RG6NBS | ABA96074 | NB_ARC (*Oryza sativa*) | 2 x 10^-08^ | R gene RPM1 (*Oryza sativa*) | 0.0011 |
| *Lp*RG7NBS | NP_001066382 | NB ARC (*Oryza sativa*) | 5 x 10^-15^ | Apoptosis ATP Binding (*Oryza sativa*) | 3.4 x 10 ^-10^ |
| *Lp*d03_gp08 | ABA92223 | NBS-LRR (*Oryza sativa*) | 2 x 10^-95^ | NBS-LRR [*Hordeum vulgare*]. | 6.1 x 10 ^-87^ |
| *Lp*d07_gp09 | ABE02736 | NBS-LRR (*Oryza sativa*) | 2 x 10^-44^ | Putative Disease (*Oryza sativa*) | 5 x 10 ^-44^ |
| *Lp*a11_gp09 | AAX19072 | resistance (*Lolium perenne*) | 1 x 10^-72^ | Disease Resistance (*Triticum aestivum*) | 5.7 x 10 ^-42^ |
| *Lp*d02_gp08 | CAG77471 | putative resistance (*Avena longiglumis*) | 8 x 10^-73^ | RGA S-217 NBS-LRR (*Hordeum vulgare)* | 6.9 x 10 ^-67^ |
| *Lp*NBS-LRR1 | BAD29107 | NBS-LRR (*Oryza sativa*) | 3 x 10 ^-51^ | NBS-LRR (*Oryza sativa*) | 8.6 x 10 ^-61^ |
| *Lp*NBS-LRR2 | BAD29107 | NBS-LRR (*Oryza sativa*) | 4 x 10^-64^ | NBS-LRR (*Oryza sativa*) | 5.5 x 10 ^-59^ |
| *Lp*NBS-LRR3 | BAC10196 | NBS-LRR (*Oryza sativa*) | 1 x 10^-07^ | NBS-LRR (*Oryza sativa*) | 1.2 x 10 ^-0.6^ |
| *Lp*NBS-LRR4 | ABA96074 | NB_ARC (*Oryza sativa*) | 8 x 10^-16^ | NB-ARC (*Oryza sativa*) | 3.5 x 10 ^-11^ |
| *Lp*NBS-LRR5 | NP_001066382 | NB ARC (*Oryza sativa*) | 1 x 10^-12^ | disease resistance (*Oryza sativa*) | 7.8 x 10 ^-11^ |
| *Lp*NBS-LRR6 | AAD46469 | HVLRR1 (*Hordeum vulgare*) | 4 x 10^-12^ | NBS-LRR (*Oryza sativa*) | 9.7 x 10^-06^ |
| *Lp*NBS-LRR7 | CAJ86357 | NB ARC (*Oryza sativa*) | 2 x 10^-31^ | ATP Binding (*Oryza sativa*) | 4.2 x 10 ^-12^ |
| *Lp*NBS-LRR8 | NP_001063004 | NBS-LRR (*Oryza sativa*) | 5 x 10^-66^ | NBS-LRR (*Oryza sativa*) | 5 x 10 ^-88^ |
| *Lp*NBS-LRR9 | CAD45034 | NBS-LRR [*Hordeum vulgare*]. | 8 x 10 ^-95^ | RGA S-L8 NBS-LRR (*Hordeum vulgare)* | 2.3 x 10^-60^ |
| *Lp*NBSC1 | EAZ40546 | Hypothetical protein(*Oryza sativa*) | 4 x 10^-58^ | Putative disease RPH8A (*Oryza sativa*) | 3.3 x 10^-58^ |
| *Lp*NBSC2 | BAC15497 | putative resistance RPH8A (*Oryza sativa*) | 2 x 10^-52^ | resistance protein RPH8A (*Oryza sativa*) | 4.2 x 10^-48^ |
| *Lp*NBSC5 | ABA96074 | NB-ARC (*Oryza sativa*) | 2 x 10^-74^ | Putative MLA1 (*Oryza sativa*) | 1.1 x 10^-69^ |
| *Lp*NBSC8 | BAC15497 | putative resistance RPH8A (*Oryza sativa*) | 4 x 10^-57^ | resistance protein RPH8A (*Oryza sativa*) | 4 x 10^-57^ |
| *Lp*NBSC15 | AAX19072 | NBS resistance protein (*Lolium perenne*) | 1 x 10^-74^ | Resistance (*Thinopirum intermedium*) | 5.9 x 10^-62^ |
| *Lp*ESTa03_10rg.1 | NP_001058528 | NB_ARC (*Oryza sativa*) | 9 x 10 ^-22^ | Putative NBS-LRR (*Oryza sativa*) | 1.4 x 10^-19^ |
| *Lp*ESTa03_10rg.2 | CAJ86357 | Rice NB_ARC (*Oryza sativa*) | 4 x 10^-41^ | Putative NBS-LRR (*Oryza sativa*) | 1.4 x 10 ^-18^ |
| *Lp*ESTa03_10rg.3 | CAJ86357 | Rice NB_ARC (*Oryza sativa*) | 3 x 10^-39^ | Putative NBS-LRR(*Oryza sativa*) | 1.8 x 10^-18^ |
| *Lp*ESTa08_14rg | BAD25211 | putative Hcr2-5B (*Oryza sativa*) | 5 x 10^-11^ | Leucine Rich Repeat (*Oryza sativa*) | 1.9 x 10^-54^ |
| *Lp*ESTa10_13rg | NP_001063004 | NBS-LRR Rice (*Oryza sativa*) | 1 x 10^-63^ | NBS-LRR (*Oryza sativa*) | 1.4 x 10 ^-86^ |
| *Lp*ESTb02_05rg | NP_001041930 | Verticcillium Wilt LRR (*Oryza sativa*) | 3 x 10^-05^ | Verticcillium Wilt LRR (*Oryza sativa*) | 6.7 x 10 ^-06^ |
| *Lp*ESTb06_11rg | NP_001067046 | Rice NB_ARC (*Oryza sativa*) | 5 x 10^-81^ | Putative NBS-LRR (*Oryza sativa*) | 1.9 x 10^-38^ |
| *Lp*ESTc10_19rg | NP_001067046 | Rice NB_ARC (*Oryza sativa*) | 3 x 10^-70^ | Putative NBS-LRR (*Oryza sativa*) | 9.1 x 10^-61^ |
| *Lp*ESTd08_13rg | BAD05298 | RPM1 (*Oryza sativa*) | 1 x 10^-29^ | RPM1 Resistance (*Oryza sativa*) | 1.5 x 10^-27^ |
| *Lp*ESTe01_10rg | ABA96074 | NB_ARC (*Oryza sativa*) | 2 x 10^-16^ | RGH2B (*Hordeum vulgare*) | 5.6 x 10^-05^ |
| *Lp*ESTe11_14rg.1 | BAD29107 | NBS-LRR like(*Oryza sativa*) | 2 x 10^-84^ | NBS-LRR (*Oryza sativa*) | 4.4 x 10 ^-58^ |
| *Lp*ESTe11_14rg.2 | BAD29107 | NBS-LRR like (*Oryza sativa*) | 2 x 10^-62^ | NBS-LRR (*Oryza sativa*) | 3 x 10 ^-60^ |
| *Lp*ESTe11_14rg.3 | CAD45024 | NBS-LRR (*Hordeum vulgare*) | 1 x 10^-57^ | RGA S-112 NBS-LRR (*Hordeum vulgare)* | 2.1 x 10 ^-61^ |
| *Lp*ESTe14_11rg.4 | NP_001048464 | Genomic DNA (*Oryza sativa*) | 1 x 10^-52^ | Phosphate transporter (*Oryza sativa*) | 2.6 x 10^-43^ |
| *Lp*ESTe14_11rg.5 | CAD45034 | NBS-LRR (*Hordeum vulgare*) | 6 x 10^-75^ | RGA S-L8 NBS-LRR (*Hordeum vulgare)* | 4.9 x 10 ^-36^ |
| *Lp*ESTe14_11rg.6 | BAD29107 | NBS-LRR (*Oryza sativa*) | 1.3 x 10 ^-87^ | NBS-LRR (*Oryza sativa*) | 2.4 x 10 ^-56^ |
| *Lp*ESTe14_11rg.7 | EAZ26793 | Serine Threonine Kinase (*Oryza sativa*) | 7 x 10^-29^ | Hypothetical protein (*Arabidopsis thaliana*) | 5.6 x 10^-08^ |
| *Lp*ESTe14_11rg.8 | ABA92223 | NBS-LRR (*Oryza sativa*) | 4 x 10^-96^ | RGA S-L8 NBS-LRR (*Hordeum vulgare)* | 1.8 x 10^-88^ |
| *Lp*ESTe14_11rg.9 | CAJ86357 | NB ARC (*Oryza sativa*) | 9 x 10^-63^ | NBS-LRR (*Oryza sativa*) | 1.7 x 10 ^-12^ |
| *Lp*ESTe14_11rg.10 | NP_001060426 | Kinase Calmodulin (*Oryza sativa*) | 2 x 10^-20^ | Protein Kinase Calmodulin (*Oryza sativa*) | 1.1 x 10 ^-13^ |
| *Lp*ESTe14_11rg.11 | NP_001046563 | NB ARC (*Oryza sativa*) | 5 x 10^-19^ | Resistance I2 (*Oryza sativa*) | 4.5 x 10 ^-58^ |
| *Lp*ESTe14_11rg.12 | NP_001066382 | NB ARC (*Oryza sativa*) | 2 x 10^-12^ | disease resistance (*Oryza sativa*) | 1 x 10 ^-10^ |
| *Lp*ESTf06_19rg.1 | NP_001043286 | NB_ARC (*Oryza sativa*) | 2 x 10^-26^ | Putative RPR1 (*Arabidopsis thaliana*) | 1.9 x 10^-24^ |
| *Lp*ESTf06_19rg.2 | NP_001043286 | NB_ARC (*Oryza sativa*) | 3 x 10^-52^ | Putative RPR1(*Arabidopsis thaliana*) | 2.9 x 10^-48^ |
| *Lp*ESTf11_11rg | NP_001049584 | NB_ARC (*Oryza sativa*) | 2 x 10^-18^ | Putative RPS2(*Arabidopsis thaliana*) | 2.9 x 10^-13^ |
| *Lp*ESTg01_20rg | BAD68095 | Verticillium wilt resistance (*Oryza sativa*) | 1 x 10^-14^ | Verticillium Wilt LRR (*Oryza sativa*) | 3.1 x 10^-13^ |
| *Lp*ESTg04_17rg.1 | CAD45034 | NBS-LRR (*Hordeum vulgare*) | 4 x 10^-74^ | RGA S-L8 NBS-LRR (*Hordeum vulgare)* | 7.4 x 10 ^-78^ |
| *Lp*ESTg04_17rg.2 | AAT69649 | putative NBS-LRR (*Oryza sativa*) | 1 x 10^-57^ | RGA S-L8 NBS-LRR (*Hordeum vulgare)* | 1.4 x 10 ^-69^ |
| *Lp*ESTg06_13rg | N/A | No significant hit | >1 | RGA S-112 NBS-LRR (*Hordeum vulgare)* | 2 x 10^-24^ |
| *Lp*ESTg10_13rg.1 | AAP54661 | Retrotransposon (*Oryza sativa*) | 1 x 10 ^-65^ | Putative resistance (*Oryza sativa*) | 9.1 x 10^-61^ |
| *Lp*ESTg10_13rg.2 | AAP54661 | Retrotransposon (*Oryza sativa*) | 3 x 10^-59^ | Rice putative resistance (*Oryza sativa*) | 6.4 x 10^-55^ |
| *Lp*ESTh04_17rg | AAT69649 | NBS-LRR (*Oryza sativa*) | 3 x 10^-62^ | NBS-LRR (*Oryza sativa*) | 1.5 x 10 ^-10^ |
| *Lp*ESTh05_28rg.1 | CAD45028 | NBS-LRR (*Hordeum vulgare*) | 1 x 10 ^-07^ | RGA S-372 NBS-LRR (*Hordeum vulgare*) | 3.6 x 10 ^-68^ |
| *Lp*ESTh05_28rg.2 | CAD45028 | NBS-LRR (*Hordeum vulgare*) | 1 x 10^-64^ | RGA S-372 NBS-LRR (*Hordeum vulgare)* | 2.3 x 10 ^-69^ |
| *Lp*ESTh07_17rg | AAT69649 | putative NBS-LRR (*Oryza sativa*) | 8 x 10^-94^ | NBS-LRR (*Oryza sativa*) | 5.1 x 10 ^-54^ |
| LPCL_38150 | AAP54661 | Retrotransposon (*Oryza sativa*) | 9 x 10^-08^ | Putative Disease (*Oryza sativa*) | 7.7 x 10 ^-08^ |
| LPCL_8913 | AAP53359 | NB ARC (*Oryza sativa*) | 0.039 | Resistance I2 (*Oryza sativa*) | 4.5 x 10 ^-58^ |
| *Lp*HvESTClone1.1 | CAD45026 | NBS-LRR (*Hordeum vulgare*) | 5 x 10^-31^ | RGA S-217 NBS-LRR (*Hordeum vulgare)* | 5.1 x 10 ^-61^ |
| *Lp*HvESTClone1.2 | CAD45026 | NBS-LRR (*Hordeum vulgare*) | 1 x 10^-66^ | RGA S-217 NBS-LRR (*Hordeum vulgare)* | 1.7 x 10 ^-42^ |
| *Lp*HvESTClone1.3 | BAD29107 | NBS-LRR (*Oryza sativa*) | 2 x 10 ^-38^ | NBS-LRR (*Oryza sativa*) | 2.5 x 10 ^-12^ |
| *Lp*HvESTClone1.4 | CAD45028 | NBS-LRR (*Hordeum vulgare*) | 6 x 10^-13^ | RG S-372 NBS-LRR (*Hordeum vulgare)* | 7.4 x 10^-69^ |
| *Lp*HvESTClone1.5 | BAD29107 | NBS-LRR (*Oryza sativa*) | 2 x 10^-74^ | NBS-LRR (*Oryza sativa*) | 3.5 x 10 ^-59^ |
| *Lp*HvESTClone2.1 | NP_001067554 | NBS-LRR (*Oryza sativa*) | 8 x 10^-47^ | NBS-LRR (*Hordeum vulgare)* | 5.4 x 10 ^-41^ |
| *Lp*HvESTClone2.2 | NP_001063004 | NBS-LRR (*Oryza sativa*) | 4 x 10 ^-64^ | NBS-LRR (*Oryza sativa*) | 1.3 x 10 ^-87^ |
| *Lp*HvESTClone3.1 | CAD45027 | NBS-LRR (*Hordeum vulgare*) | 4 x 10^-52^ | RGA S-226 NBS-LRR (*Hordeum vulgare)* | 2.2 x 10 ^-48^ |
| *Lp*HvESTClone3.2 | NP_001043286 | NB_ARC (*Oryza sativa*) | 5 x 10^-20^ | HV1LRR (*Hordeum vulgare)* | 1 x 10 ^-16^ |
| *Lp*HvESTClone4.1 | CAD45030 | NBS-LRR (*Hordeum vulgare*) | 9 x 10^-52^ | RG S-9202 NBS-LRR (*Hordeum vulgare)* | 3.7 x 10 ^-60^ |
| *Lp*HvESTClone4.2 | BAD29107 | NBS-LRR (*Oryza sativa*) | 2 x 10^-62^ | NBS-LRR (*Oryza sativa*) | 8.6 x 10 ^-61^ |
| *Lp*HvESTClone4.3 | AAT69649 | NBS-LRR (*Oryza sativa*) | 1 x 10 ^-59^ | NBS-LRR (*Oryza sativa*) | 2.4 x 10 ^-47^ |
| *Lp*HvESTClone4.4 | BAC10196 | NBS-LRR (*Oryza sativa*) | 9 x 10^-14^ | NBS-LRR (*Oryza sativa*) | 2.1 x 10 ^-12^ |
| *Lp*HvESTClone4.5 | ABA96074 | NB_ARC (*Oryza sativa*) | 4 x 10^-32^ | RHGB2 (*Hordeum vulgare*) | 1.1 x 10 ^-07^ |
| *Lp*AG205017 | BAC55682 | Putative RPR1 (*Oryza sativa*) | 8 x 10^-50^ | **NB-ARC protein** (*Oryza sativa*) | 2.4 x 10^-22^ |
| *Lp*AG205018 | ABU46265 | NBS-LRR (*Musa x paradisiacal*) | 8 x 10^-26^ | **Uncharacterized protein** (*Oryza sativa*) | 5.3 x 10^-52^ |
| *Lp*AG205035 | AAP45181 | RG3 protein (*Solanum bulbocastanum*) | 4 x 10^-45^ | **Uncharacterized protein** (*Oryza sativa*) | 4.0 x 10^-52^ |
| *Lp*AG205050 | ABA95301 | NB-ARC (*Oryza sativa*) | 4 x 10^-19^ | **Uncharacterized protein**(*Oryza sativa*) | 5.2 x 10^-17^ |
| *Lp*AG205055 | BAC55682 | putative RPR1 (*Oryza sativa*) | 2 x 10^-54^ | **OSJNBa0059D20.15 protein** (*Oryza sativa*) | 9.0 x 10^-44^ |
| *Lp*AG205063 | AAC31553 | NBS-LRR protein O2 (*Avena sativa*) | 2 x 10^-25^ | NBS-LRR protein O2 (*Avena sativa*) | 2.4 x 10^-25^ |
