# Supplementary material for: Molecular characterisation and genetic mapping of candidate genes for qualitative disease resistance in perennial ryegrass (Lolium perenne L.)
Source: BMC Plant Biol. 2009 May 19;9:62. doi: 10.1186/1471-2229-9-62 (PMC2694799; doi:10.1186/1471-2229-9-62)
Supplement: Additional File 3 — Summary details for specific R gene-directed degenerate primer pair combinations, as described in Additional File 1, along with primer pair code, numbers of amplification products and corresponding R gene templates. [file 1471-2229-9-62-S3.doc]

**Additional File 3**

| **Primer pair combination** | **Primer pair combination code** | **Number of amplification products generated** | **R genes amplified** |
| --- | --- | --- | --- |
| PUBDegs1/PUBDegas1 | A | 1 | *Lp*d03_gp08  *Lp*d07_gp09  *Lp*NBSC1  *Lp*NBSC2 |
| PUBDegs1/PUBDegas2 | B | 1 | *Lp*a11_gp09 |
| PUBDegs1/PUBDegas3 | C | 1 | *Lp*d02_gp08  *Lp*NBS-LRR6  *Lp*NBSC8 |
| DEGH2018SbF/DEGLi*etal.*(2006)R3 | D | 1 | *Lp*RG1NBS  *Lp*RG2NBS  *Lp*RG3NBS  *Lp*RG4NBS  *Lp*RG5NBS  *Lp*RG6NBS  *Lp*RG7NBS |
| DEGBHvF1/DEGGLHvR2 | E | 1 | *Lp*RGContig2 |
| DEGBHvF1/DEGH202 | F | 2 | *Lp*RGContig1 |
| DEGBHvF1/ DEGLi*etal*.(2006)R3 | G | 2 | *Lp*NBSC15  *Lp*RGContig3 |
| DEGLi*etal*.(2006)F1/DEGLi*etal*.(2006)R3 | H | 2 | *Lp*NBS-LRR1  *Lp*NBS-LRR2  *Lp*NBS-LRR3  *Lp*NBS-LRR4  *Lp*NBS-LRR5  *Lp*NBS-LRR6  *Lp*NBS-LRR7  *Lp*NBS-LRR8  *Lp*NBS-LRR9 |
