# Supplementary material for: Molecular characterisation and genetic mapping of candidate genes for qualitative disease resistance in perennial ryegrass (Lolium perenne L.)
Source: BMC Plant Biol. 2009 May 19;9:62. doi: 10.1186/1471-2229-9-62 (PMC2694799; doi:10.1186/1471-2229-9-62)
Supplement: Additional File 4 — Functional characterisation of predicted translation products from perennial ryegrass candidate R genes. All information was derived from Pfam links within the best-available Uniprot wuBLASTX hits in BASC. Pfam information was used to obtain the probable location of candidate sequences, protein size, position of NBS sequence and number of LRR repeats. [file 1471-2229-9-62-S4.doc]

**Additional File 4**

| **Perennial ryegrass unique identifier (UI)** | **wuBLASTX-based functional annotation (in BASC system)** | **Position of the NBS domain** | **Pfam reference of UI (Domain location of UI with respect to wuBLASTX protein match)** | **Protein size (amino acids)** | **Number of LRR repeats in highest Pfam**  **match** | **Type of R gene** |
| --- | --- | --- | --- | --- | --- | --- |
| *Lp*Lrk10.1 | **Receptor kinase LRK14 *(Avena sativa)*** | N/A | Protein kinase | 639 | 0 | Protein kinase |
| *Lp*Lrk10.2 | Protein Kinase Calmodulin (*Oryza sativa*) | N/A | Protein kinase | 1238 | 0 | Kinase |
| *Lp*PcaClone1.1 | NBS_LRR 1 (*Avena strigosa*) | 8-167 | NBS | 179 | 0 | NBS |
| *Lp*PcaClone1.2 | NBS_LRR 3(*Avena strigosa*) | 8-167 | NBS | 179 | 0 | NBS |
| *Lp*PcaClone2.1 | NBS_LRR 3(*Avena strigosa*) | 8-167 | NBS | 179 | 0 | NBS |
| *Lp*PcaClone2.2 | NBS_LRR 3(*Avena strigosa*) | 8-167 | NBS | 179 | 0 | NBS |
| *Lp*PcaClone3.1 | Putative R-gene (*Avena strigosa*) | 49-167 | NBS | 179 | 0 | NBS |
| *Lp*PcaClone3.2 | Putative R-gene (*Avena strigosa*) | 1-105 | NBS | 105 | 0 | NBS |
| *Lp*PcaClone3.3 | Putative R-gene (*Avena strigosa*) | 1-179 | NBS | 179 | 0 | NBS |
| *Lp*PcaClone4.1 | Putative R-gene (*Avena strigosa*) | 8-167 | NBS | 179 | 0 | NBS |
| *Lp*PcaClone4.2 | Putative R-gene (*Avena strigosa*) | 8-167 | NBS | 179 | 0 | NBS |
| *Lp*PcaClone4.3 | NBS_LRR 3 (*Avena strigosa*) | 8-167 | NBS | 179 | 0 | NBS |
| *Lp*HvClone1 | NBS-LRR S-120 (*Hordeum vulgare)* | 10 102 | N terminus of NBS | 179 | 0 | NBS |
| *Lp*HvClone2 | RGA S-215 NBS-LRR (*Hordeum vulgare)* | 8-92 | NBS | 181 | 0 | NBS |
| *Lp*HvClone3 | RGA S-215 NBS-LRR (*Hordeum vulgare)* | 8-92 | NBS | 181 | 0 | NBS |
| *Lp*HvClone4 | RGA S-215 NBS-LRR (*Hordeum vulgare)* | 8-92 | NBS | 181 | 0 | NBS |
| *Lp*HvClone5 | RGA S-215 NBS-LRR (*Hordeum vulgare)* | 8-92 | NBS | 181 | 0 | NBS |
| *Lp*RGcontig1 | Resistance (*Thinopirum intermedium*) | 1-180 | NBS | 180 | 0 | NBS |
| *Lp*RGcontig2 | Putative disease RPH8A (*Oryza sativa*) | 784-915 | NBS | 1377 | 2 | NBS-NBS-LRR |
| *Lp*RGcontig3 | NBS-LRR disease (*Oryza sativa*) | 315-486 | NBS | 1521 | 4 | NBS-LRR |
| *Lp*RG1NBS | NBS-LRR like Rice (*Oryza sativa*) | 1-176 | NBS | 176 | 0 | NBS |
| *Lp*RG2NBS | NBS-LRR like Rice (*Oryza sativa*) | 1-172 | NBS | 176 | 0 | NBS |
| *Lp*RG3NBS | Protein Kinase Calmodulin (*Oryza sativa*) | 542-594 | N/A | 1238 | 0 | Kinase |
| *Lp*RG4NBS | Putative resistance (*Oryza sativa*) | 92-264 | N terminus of NBS | 930 | 2 | CC-NBS-LRR |
| *Lp*RG5NBS | RHGB2 (*Hordeum vulgare*) | 720-978 | NBS | 1021 | 0 | NBS |
| *Lp*RG6NBS | R gene RPM1 (*Oryza sativa*) | 720-978 | NBS | 1021 | 0 | NBS |
| *Lp*RG7NBS | NBS-LRR (*Oryza sativa*) | 1-270 | NBS | 430 | 0 | NBS |
| *Lp*d03_gp08 | NBS-LRR [*Hordeum vulgare*]. | 154-372 | NBS | 909 | 3 | NBS-LRR |
| *Lp*d07_gp09 | Putative Disease (*Oryza sativa*) | 198-377 | NBS | 1014 | 0 | NBS |
| *Lp*a11_gp09 | Disease Resistance (*Triticum aestivum*) | 198-377 | NBS | 1014 | 0 | NBS |
| *Lp*d02_gp08 | RGA S-217 NBS-LRR (*Hordeum vulgare)* | 4-161 | NBS | 163 | 0 | NBS |
| *Lp*NBS-LRR1 | NBS-LRR (*Oryza sativa*) | 238-405 | NBS | 581 | 3 | CC-NBS-LRR |
| *Lp*NBS-LRR2 | NBS-LRR (*Oryza sativa*) | 238-405 | NBS | 581 | 3 | CC-NBS-LRR |
| *Lp*NBS-LRR3 | NBS-LRR (*Oryza sativa*) | 164-446 | 3’ of NBS | 928 | 0 | NBS |
| *Lp*NBS-LRR4 | NB-ARC (*Oryza sativa*) | 810-974 | C terminus | 1021 | 0 | NBS |
| *Lp*NBS-LRR5 | Apoptosis ATP Binding (*Oryza sativa*) | 156-293 | N-terminus and NBS | 924 | 3 | CC-NBS-LRR |
| *Lp*NBS-LRR6 | NBS-LRR (*Oryza sativa*) | 674-852 | C terminus- LRR2 | 905 | 2 | CC-CC-NBS-LRR |
| *Lp*NBS-LRR7 | ATP Binding (*Oryza sativa*) | 263-378 | N terminus and NBS | 2113 | 3 | CC-NBS-LRR |
| *Lp*NBS-LRR8 | NBS-LRR (*Oryza sativa*) | 310-522 | NBS | 736 | 2 | NBS-LRR |
| *Lp*NBS-LRR9 | RGA S-L8 NBS-LRR (*Hordeum vulgare)* | 226-420 | NBS | 905 | 0 | NBS AAA |
| *Lp*NBSC1 | Putative disease RPH8A (*Oryza sativa*) | 729-915 | NBS | 1377 | 2 | NBS-NBS-LRR |
| *Lp*NBSC2 | Resistance protein RPH8A (*Oryza sativa*) | 726-911 | NBS | 1377 | 2 | NBS-NBS-LRR |
| *Lp*NBSC5 | Putative MLA1 (*Oryza sativa*) | 273-510 | NBS | 898 | 2 | CC-NBS-LRR |
| *Lp*NBSC8 | resistance protein RPH8A (*Oryza sativa*) | 164-446 | NBS | 1266 | 0 | NBS |
| *Lp*NBSC15 | Resistance (*Thinopirum intermedium*) | 1-180 | NBS | 180 | 0 | NBS |
| *Lp*ESTa03_10rg.1 | Putative NBS-LRR (*Oryza sativa*) | 222-352 | NBS | 1494 | 3 | NBS-LRR |
| *Lp*ESTa03_10rg.2 | Putative NBS-LRR (*Oryza sativa*) | 222-352 | NBS | 1494 | 3 | NBS-LRR |
| *Lp*ESTa03_10rg.3 | Putative NBS-LRR (*Oryza sativa*) | 222-352 | NBS | 1494 | 3 | NBS-LRR |
| *Lp*ESTa08_14rg | Leucine Rich Repeat (*Oryza sativa*) | N/A | LRR | 956 | 12 | LRR protein |
| *Lp*ESTa10_13rg | NBS-LRR (*Oryza sativa*) | 313-513 | NBS- | 905 | 2 | NBS-LRR |
| *Lp*ESTb02_05rg | Verticcillium Wilt LRR (*Oryza sativa*) | 367-588 | LRR 7-13 | 1062 | 23 | LRR |
| *Lp*ESTb06_11rg | Putative NBS-LRR (*Oryza sativa*) | 580-805 | NBS | 961 | 0 | CC-NBS |
| *Lp*ESTc10_19rg | LR10 (*Oryza sativa*) | 456-635 | LRR1-2 | 947 | 2 | CC-NBS-NBS-LRR |
| *Lp*ESTd08_13rg | RPM1 Resistance (*Oryza sativa*) | 1-93 | N terminus | 909 | 3 | NBS-LRR |
| *Lp*ESTe01_10rg | RGH2B (*Hordeum vulgare*) | 810-974 | C terminus | 1021 | 0 | NBS |
| *Lp*ESTe11_14rg.1 | Putative RPR1 (*Arabidopsis thaliana*) | 674-852 | C terminus- LRR2 | 905 | 2 | CC-CC-NBS-LRR |
| *Lp*ESTe11_14rg.2 | Putative RPR1(*Arabidopsis thaliana*) | 674-852 | C terminus- LRR2 | 905 | 2 | CC-CC-NBS-LRR |
| *Lp*ESTe11_14rg.3 | RG S-112 NBS-LRR (*Hordeum vulgare)* | 226-420 | NBS | 581 | 0 | NBS AAA |
| *Lp*ESTe14_11rg.4 | Phosphate transporter (*Oryza sativa*) | 665-742 | EXS | 707 | 0 | SPX-EXS |
| *Lp*ESTe14_11rg.5 | RGA S-L8 NBS-LRR (*Hordeum vulgare)* | 572-732 | 3 LRRS | 909 | 0 | NBS-LRR |
| *Lp*ESTe14_11rg.6 | NBS-LRR (*Oryza sativa*) | 238-405 | NBS | 581 | 3 | CC-NBS-LRR |
| *Lp*ESTe14_11rg.7 | Hypothetical protein (*Arabidopsis thaliana*) | 886-971 | Kinase Tys | 963 | 6 | Kinase Tyr |
| *Lp*ESTe14_11rg.8 | Uncharacterized protein (*Oryza sativa*) | 270-370 | NBS | 397 | 0 | NBS |
| *Lp*ESTe14_11rg.9 | NBS-LRR (*Oryza sativa*) | 223-327 | N terminus and NBS | 1492 | 2 | NBS-LRR |
| *Lp*ESTe14_11rg.10 | Resistance I2 (*Oryza sativa*) | 487-679 | LRR 1-LRR2 | 1320 | 3 | NBS-LRR |
| *Lp*ESTe14_11rg.11 | Resistance I2 (*Oryza sativa*) | 487-679 | LRR 1-LRR2 | 1320 | 3 | NBS-LRR |
| *Lp*ESTe14_11rg.12 | Disease resistance (*Oryza sativa*) | 156-293 | N-terminus of NBS | 924 | 3 | CC-NBS-LRR |
| *Lp*ESTf06_19rg.1 | Putative RPR1 (*Arabidopsis thaliana*) | 674-852 | C terminus- LRR2 | 905 | 2 | CC-CC-NBS-LRR |
| *Lp*ESTf06_19rg.2 | Putative RPR1(*Arabidopsis thaliana*) | 674-852 | C terminus- LRR2 | 905 | 2 | CC-CC-NBS-LRR |
| *Lp*ESTf11_11rg | Putative RPS2(*Arabidopsis thaliana*) | 330-515 | C terminus -NBS | 975 | 3 | CC-CC-NBS-LRR |
| *Lp*ESTg01_20rg | Verticillium Wilt LRR (*Oryza sativa*) | 923-1023 | C terminus and LRR21 | 1049 | 21 | LRR protein |
| *Lp*ESTg04_17rg.1 | RGA S-L8 NBS-LRR (*Hordeum vulgare)* | 562-779 | LRR 1,2 and 3 | 909 | 3 | NBS-LRR |
| *Lp*ESTg04_17rg.2 | RGA S-L8 NBS-LRR (*Hordeum vulgare)* | 572-732 | 3 LRRS | 909 | 3 | NBS-LRR |
| *Lp*ESTg06_13rg | RGA S-112 NBS-LRR (*Hordeum vulgare)* | 240-460 | NBS | 702 | 0 | NBS |
| *Lp*ESTg10_13rg.1 | Putative resistance (*Oryza sativa*) | 417-586 | N terminus AAA eve | 921 | 0 | eve AAA Chr |
| *Lp*ESTg10_13rg.2 | Putative resistance (*Oryza sativa*) | 417-586 | N terminus AAA eve | 921 | 0 | eve AAA Chr |
| *Lp*ESTh04_17rg | NBS-LRR (*Oryza sativa*) | 861-1065 | LRR3 and C terminus | 1081 | 3 | CC-CC-NBS-LRR |
| *Lp*ESTh05_28rg.1 | RGA S-372 NBS-LRR (*Hordeum vulgare*) | 154-372 | NBS | 909 | 3 | CC-NBS-LRR |
| *Lp*ESTh05_28rg.2 | RGA S-372 NBS-LRR (*Hordeum vulgare)* | 154-372 | NBS | 909 | 3 | NBS-LRR |
| *Lp*ESTh07_17rg | NBS-LRR (*Oryza sativa*) | 154-372 | NBS | 909 | 3 | CC-NBS-LRR |
| LPCL_38150 | Putative Disease (*Oryza sativa*) | 654-873 | rve CC Chromo | 921 | 0 | rve CC Chr |
| LPCL_8913 | Resistance I2 (*Oryza sativa*) | 487-679 | LRR 1-2 | 1320 | 3 | NBS-LRR |
| *Lp*HvESTClone1.1 | RGA S-217 NBS-LRR (*Hordeum vulgare)* | 1410-1605 | C terminus of NBS | 1622 | 0 | NBS |
| *Lp*HvESTClone1.2 | RGA S-217 NBS-LRR (*Hordeum vulgare)* | 1410-1605 | C terminus of NBS | 1622 | 0 | NBS |
| *Lp*HvESTClone1.3 | NBS-LRR (*Oryza sativa*) | 238-405 | NBS | 581 | 3 | CC-NBS-LRR |
| *Lp*HvESTClone1.4 | RGA S-372 NBS-LRR (*Hordeum vulgare)* | 447-629 | NBS | 1366 | 2 | NBS-LRR |
| *Lp*HvESTClone1.5 | NBS-LRR (*Oryza sativa*) | 238-405 | NBS | 581 | 3 | CC-NBS-LRR |
| *Lp*HvESTClone2.1 | NBS-LRR (*Hordeum vulgare)* | 143-314 | N terminus of NBS | 962 | 1 | CC-NBS-LRR |
| *Lp*HvESTClone2.2 | NBS-LRR (*Oryza sativa*) | 313-513 | NBS- | 581 | 2 | NBS-LRR |
| *Lp*HvESTClone3.1 | RGA S-226 NBS-LRR (*Hordeum vulgare)* | 1371-1578 | LRR 1 and 2 | 1579 | 2 | NBS-LRR |
| *Lp*HvESTClone3.2 | HV1LRR (*Hordeum vulgare)* | 683-828 | Cterminus | 865 | 3 | CC-NBS-LRR |
| *Lp*HvESTClone4.1 | RGA S-9202 NBS-LRR (*Hordeum vulgare)* | 1-205 | N terminus and NBS | 1014 | 0 | NBS |
| *Lp*HvESTClone4.2 | NBS-LRR (*Oryza sativa*) | 238-405 | NBS | 581 | 3 | CC-NBS-LRR |
| *Lp*HvESTClone4.3 | NBS-LRR (*Oryza sativa*) | 861-1054 | LRR 3 and C terminus | 581 | 3 | CC-CC-NBS-LRR |
| *Lp*HvESTClone4.4 | Disease resistance (*Oryza sativa*) | 92-264 | N terminus of NBS | 930 | 2 | CC-NBS-LRR |
| *Lp*HvESTClone4.5 | RHGB2 (*Hordeum vulgare*) | 720-978 | NBS | 1021 | 0 | NBS |
| *Lp*AG205017 | **Uncharacterized protein** (*Oryza sativa*) | 194-465 | NBS | 927 | 2 | NBS-LRR |
| *Lp*AG205018 | **Uncharacterized protein** (*Oryza sativa*) | 179-472 | NBS | 1076 | 0 | NBS |
| *Lp*AG205035 | **Uncharacterized protein** (*Oryza sativa*) | 151-427 | NBS | 1264 | 6 | NBS-LRR |
| *Lp*AG205050 | **Uncharacterized protein** (*Oryza sativa*) | 182-465 | NBS | 1004 | 0 | NBS |
| *Lp*AG205055 | **OSJNBa0059D20.15 protein** (*Oryza sativa*) | 238-506 | NBS | 740 | 0 | NBS |
| *Lp*AG205063 | NBS-LRR protein O2 (*Avena sativa*) | 1-238 | NBS | 456 | 1 | NBS-LRR |

^a^All information was derived from Pfam links within the best available Uniprot wuBLASTX hits in BASC. Pfam information was used to obtain the probable location of candidate sequences, protein size, position of NBS sequence and the number of LRR repeats.
