# Supplementary material for: Molecular characterisation and genetic mapping of candidate genes for qualitative disease resistance in perennial ryegrass (Lolium perenne L.)
Source: BMC Plant Biol. 2009 May 19;9:62. doi: 10.1186/1471-2229-9-62 (PMC2694799; doi:10.1186/1471-2229-9-62)
Supplement: Additional File 5 — Major protein sequence motifs in predicted Lolium NBS domains. aMotifs listed in the order of occurrence in the NBS domain of putative perennial ryegrass R genes. Perennial ryegrass motifs were named in accordance with descriptions obtained from both rice and A. thaliana [30,31,66]; bBioinformatic analysis using Pfam on putative R gene sequences identified all to be CC-NBS types (CNL denotes CC-NBS-LRR and TNL denotes TIR-NBS-LRR); cConsensus amino acid sequences for Lolium NBS sequences were derived from MEME, while those for wheat were derived from [58]. [file 1471-2229-9-62-S5.doc]

**Additional File 5**

| **Motif^a^** | **Group^b^** | **Consensus sequence^c^** | **Source species** |
| --- | --- | --- | --- |
| P-Loop  P-Loop  P-Loop  P-Loop | CNL  CNL  CNL  CNL | GGMGKTTLAMYVYQ  GGxGKTTLAQxVY  GMGGLGKTTLAQxVYN  GMGGVGKTTLARQIF | Perennial ryegrass  Wheat  Rice  *Arabidopsis* |
| RNBS-I | CNL | FQCAAWVTVSQTFTV | Perennial ryegrass |
| RNBS-I  RNBS-I  RNBS-A  RNBS-A | CNL  CNL  CNL  TNL | FDCRAWVHVSKxFSV  FDCRAWVCVSQNFDVXKLLR  VKxGFDIVIWVVVSQEFTLKKIQQDILEK  DYGMKLHLQEQFLSEILNQKDIKIxHLGV | Wheat  Rice  *Arabidopsis*  *Arabidopsis* |
| Kin-2A | CNL | KRFLLVLDDVWEF | Perennial ryegrass |
| Kin-2A | CNL | KRFLLVLDDVW | Wheat |
| Kin-2A | CNL | KRFLLVLDDV | Rice |
| Kin-2A | CNL | KRFLLVLDDIW | *Arabidopsis* |
| Kin-2A | TNL | KKDKKVLIVLDDVD | *Arabidopsis* |
| RNBS-II  RNBS-II  RNBS-II  RNBS-B  RNBS-C  RNBS-C  RNBS-III  RNBS-C | CNL  CNL  CNL  CNL  CNL  CNL  CNL  CNL | GSRIIVTTRIExVAx  GSRILVTTR  GSRIIVTTRIExVAx  NGCKVLFTTRSEEVC  YKLEPLSDDDSWxLF  LDDSWxLF  VECLTPEEAWELFQRKV  KVECLTPEEAWELFQRKV | Perennial ryegrass  Wheat  Rice  *Arabidopsis*  Perennial ryegrass  Wheat  Rice  *Arabidopsis* |
| GLPL  GLPL  GLPL  GLPL | CNL  CNL  CNL  CNL | IVNKCGGLPLA  IxxKCGGLPLA  ILKKCGGLPLA  VAKKCGGLPLA | Perennial ryegrass  Wheat  Rice  *Arabidopsis* |
|  |  |  |  |
| RNBS-V  RNBS-V  RNBS-V  RNBS-D  RNBS-D | CNL  CNL  CNL  CNL  TNL | IFPKDYEIDVD  N/A  KQCFLYCSIFPEDYxIxRDxLIRLWIAEGFI  CFLYCALFPEDYEIxKEKLIDYWIAEGFI  EDKDLFLHIACFFNG | Perennial ryegrass  Wheat  Rice  *Arabidopsis*  *Arabidopsis* |
