# Supplementary material for: Molecular characterisation and genetic mapping of candidate genes for qualitative disease resistance in perennial ryegrass (Lolium perenne L.)
Source: BMC Plant Biol. 2009 May 19;9:62. doi: 10.1186/1471-2229-9-62 (PMC2694799; doi:10.1186/1471-2229-9-62)
Supplement: Additional File 6 — Summary information of amino acid structure for NBS domain protein sequence motifs numbered in Fig. 2, based on matching using the MEME program. [file 1471-2229-9-62-S6.doc]

**Additional File 6**

| **Motif number** | **Motif size**  **(amino acids)** | **Consensus Peptide Structure** |
| --- | --- | --- |
| 1  2  3  4  5  6  7  8  9  10  11  12  13  14  15  16  17  18  19  20  21  22  23  24  25  26  27  28  29  30  31  32  33  34  35  36  37  38  39  40  41  42  43  44  45  46  47  48  49  50 | 15  15  15  15  15  15  15  15  15  15  15  15  15  15  15  15  15  15  11  15  15  15  15  15  15  15  14  11  11  11  11  15  15  15  15  15  15  15  11  15  15  15  11  15  15  15  8  15  8  11 | YNDLPSHMKQCFAFC  YENLPDERTKHCFLF  TQYIYNNKQVQDHFQ  CVYVPYAINFWMGHG  DKGVAEIMGTVKAYN  EIVERCAGSPLAATA  EEEWKAVSRRSNICT  QHVCHRMQAHGQVIM  LQHGGMGSVILTTTR  GQAMASKESEHEWKH  QKEEERPTVLVNMVG  KRFLLVLDDVWEFTD  TVIPIVGPGGIGKTT  SQFHKVPDAESNLFH  YLRGHSVLGCLKRAC  PIGPQNRQMVVITTR  YAKSIVNMCGGLPLA  LEDEFIKEIIETRAF  WVCVSLDFNAN  YLLVLDDVWTHHYWE  RRFLLLIDDLWQMLD  FEEAWNLFDANAGCN  FQCAAWVTVSQTFTV  QGSRIIITTRSQHVA  KGSPLAAATLGRILR  PTLYGMDPQKNTVIE  GGMGKTTLAMYVYQ  IFPKDYEIDVD  ESGILPILKLS  WKREVDKWEQL  LPEEHPRGESY  QVDNEQDGYYPDLIE  LPEDMFWEFFCACAF  SYVRVMQNHVAYFQK  HYEGHPELQPIGKHI  NGCMILVTTRMPDVA  CAMVSTILGLEFSGS  FFKRLFGNRDNCPTV  FIDEDGDVRNN  HVCKDTQEYENMSNF  AACVTMTCTTSNALE  LQMWVCVSTDFNERR  LVQIGIPQGGR  FLQSDRSNESVFGLW  YDYLPFDLRKCFSYF  ACCLREHDKAYDIEP  GSIMRNKT  FDREHWGWIAGKHEW  TNSSHQIR  TPIRARQIVPR |
