# Supplementary material for: Molecular characterisation and genetic mapping of candidate genes for qualitative disease resistance in perennial ryegrass (Lolium perenne L.)
Source: BMC Plant Biol. 2009 May 19;9:62. doi: 10.1186/1471-2229-9-62 (PMC2694799; doi:10.1186/1471-2229-9-62)
Supplement: Additional File 7 — Reference information for sequences corresponding to individual clusters identified during phylogenetic analysis for the complete NBS domain (P-Loop-GLPL), as depicted in Additional File 8. [file 1471-2229-9-62-S7.doc]

**Additional File 7**

| **Dendrogram cluster code** | **Sequence unique identifier** | **Species of origin** | **Reference** |
| --- | --- | --- | --- |
|  |  |  |  |
| A | NBS Hordeum | *H. vulgare* | [38] |
|  | *Lp*RGContig3 | *L. perenne* | This study |
|  | RPS2Arabidopsis | *A. thaliana* | [38] |
| B | PRFTomato | *Lycopersicon esculentum* | [38] |
|  | *Lp*NBSC15 | *L. perenne* | This study |
|  | *Lp*a11_gp09 | *L. perenne* | This study |
|  | AY923221.1 | *Lolium spp.* | [38] |
|  | AY923216.1 | *Lolium spp.* | [38] |
|  | AY923218.1 | *Lolium spp.* | [38] |
| C | *Lp*HvESTClone2.1 | *L. perenne* | This study |
|  | NBSRice | *Oryza sativa* | [38] |
|  | *Lp*d03_gp08 | *L. perenne* | This study |
|  | *Lp*NBSC10 | *L. perenne* | This study |
| D | *Lp*RGContig2 | *L. perenne* | This study |
|  | NBSRPH1Rice | *Oryza sativa* | [38] |
|  | *Lp*NBSC8 | *L. perenne* | This study |
|  | *Lp*NBSC1 | *L. perenne* | This study |
|  | *Lp*NBSC2 | *L. perenne* | This study |
| E | RGC2BLettuce | *Lactuca sativa* | [38] |
|  | Arabidopsis | *A. thaliana* | [38] |
|  | L6Flax | *Linum usitatissium* | [38] |
|  | MFlax | *Linum usitatissium* | [38] |
| F | AY932219.1 | *Lolium spp.* | [38] |
|  | *Lp*d02_gp08 | *L. perenne* | This study |
|  | *Lp*NBSC5 | *L. perenne* | This study |
|  | *Lp*RG2NBS | *L. perenne* | This study |
|  | *Lp*RG1NBS | *L. perenne* | This study |
|  | AY923228.1 | *Lolium spp.* | [38] |
| G | AY923215.1 | *Lolium spp.* | [38] |
|  | I2C-1Tomato | *Lycopersicon esculentum* | [38] |
|  | AY923217.1 | *Lolium spp.* | [38] |
|  | DQ010112.1 | *Lolium spp.* | [38] |
|  | AY923223.1 | *Lolium spp.* | [38] |
|  | AY923222.1 | *Lolium spp.* | [38] |
|  | AY923227.1 | *Lolium spp.* | [38] |
|  | AY923220.1 | *Lolium spp.* | [38] |
|  | AY923224.1 | *Lolium spp.* | [38] |
|  |  |  |  |
