# Supplementary figures and images for: Molecular characterisation and genetic mapping of candidate genes for qualitative disease resistance in perennial ryegrass (Lolium perenne L.)
Source: BMC Plant Biol. 2009 May 19;9:62. doi: 10.1186/1471-2229-9-62 (PMC2694799; doi:10.1186/1471-2229-9-62)

## Slide 1
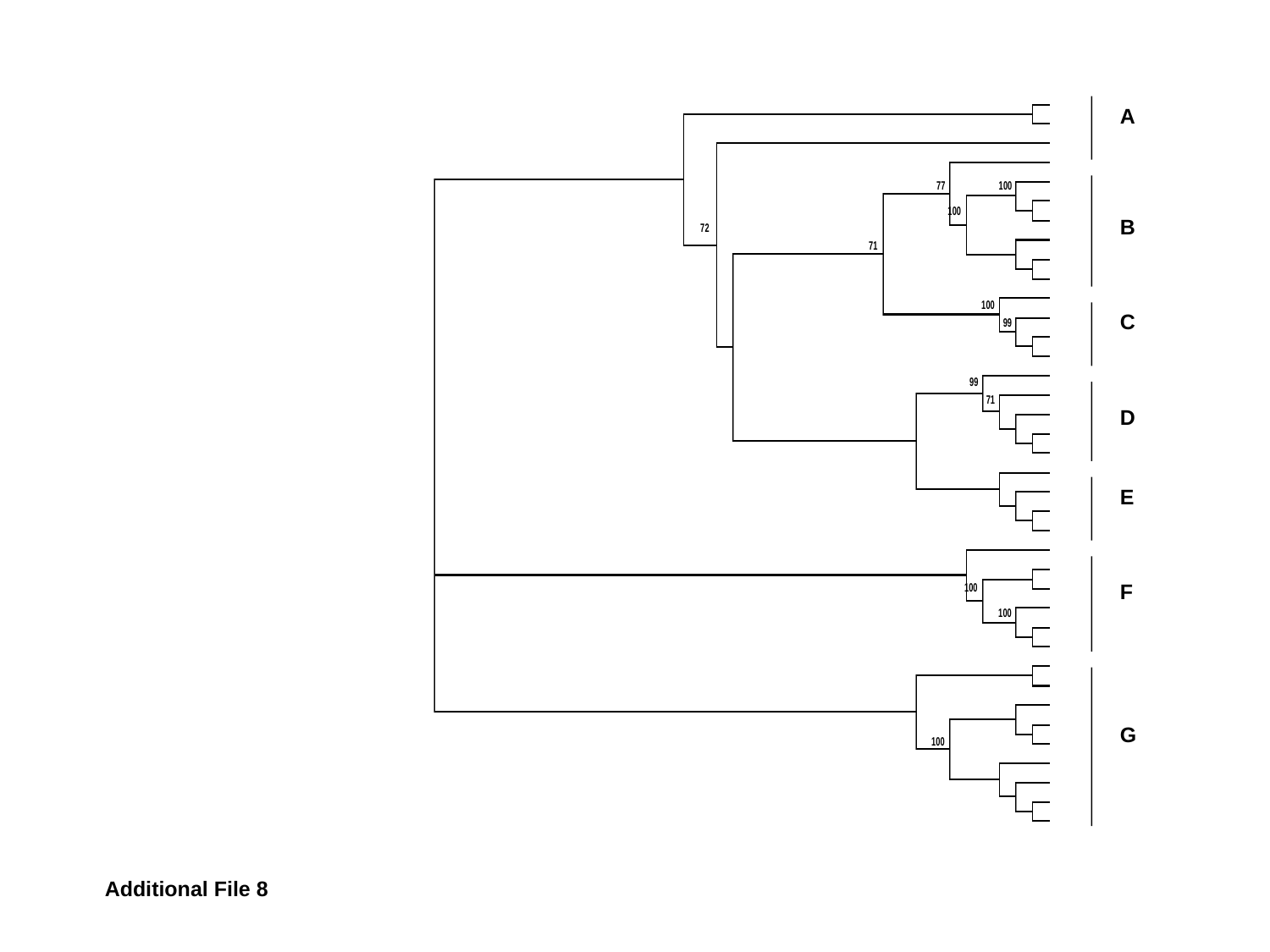

A
B
C
D
E
F
G
Additional File 8

Supplement: Additional File 8 — NJ dendrograms based on amino acid alignment of the full-length (P-Loop – GLPL) regions of NBS protein domains encoded by Lolium R genes. Bootstrap values are displayed as percentages of 1000 neighbour joining bootstrap replications. Bootstrap values at or greater than 65% are shown. Bars at the right of the dendrograms represent R gene sub-classes. [file 1471-2229-9-62-S8.ppt]

## Slide 1
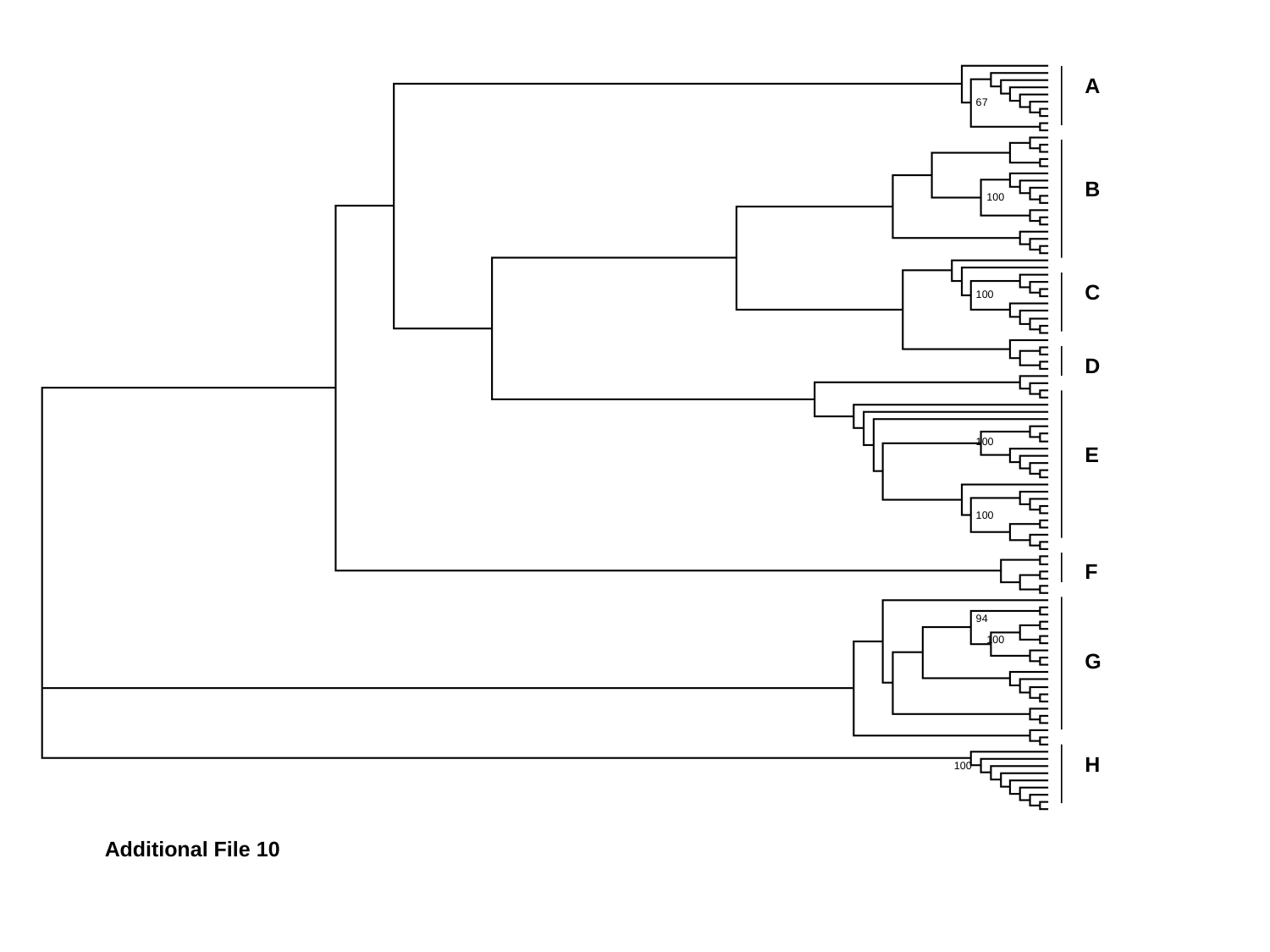

A
67
B
100
C
100
D
100
E
100
F
94
100
G
H
100
Additional File 10

Supplement: Additional File 10 — NJ dendrograms based on amino acid alignment of the partial (Kin-2A -GLPL) regions of NBS protein domains encoded by Lolium R genes. Details are as described in the legend for Additional File 8. [file 1471-2229-9-62-S10.ppt]
