# Supplementary material for: Molecular characterisation and genetic mapping of candidate genes for qualitative disease resistance in perennial ryegrass (Lolium perenne L.)
Source: BMC Plant Biol. 2009 May 19;9:62. doi: 10.1186/1471-2229-9-62 (PMC2694799; doi:10.1186/1471-2229-9-62)
Supplement: Additional File 9 — Reference information for sequences corresponding to individual clusters identified during phylogenetic analysis for the Kin-2A-GLPL region of the NBS domain, as depicted in Additional File 10. [file 1471-2229-9-62-S9.doc]

**Additional File 9**

| **Dendrogram cluster code** | **Sequence unique identifier** | **Species of origin** | **Reference** |
| --- | --- | --- | --- |
| A | *As*PcaClone5 | *Avena sativa* | [37] |
|  | AG205005 | *L. multiflorum* | [39] |
|  | *Lp*PcaClone4.1 | *L. perenne* | This study |
|  | AG205010 | *L. multiflorum* | [39] |
|  | *Lp*PcaClone1.1 | *L. perenne* | This study |
|  | *As*PcaClone 5 | *Avena sativa* | [37] |
|  | *Lp*PcaClone3.3 | *L. perenne* | This study |
|  | AG205006 | *L. multiflorum* | [39] |
|  | AG205059 | *L. multiflorum* | [39] |
| B | AG205012 | *L. multiflorum* | [39] |
|  | AG205043 | *L. multiflorum* | [39] |
|  | AG205048 | *L. multiflorum* | [39] |
|  | AG205040 | *L. multiflorum* | [39] |
|  | AG205046 | *L. multiflorum* | [39] |
|  | AG205044 | *L. multiflorum* | [39] |
|  | NBSRice | *Oryza sativa* | [38] |
|  | *Lp*NBSC10 | *L. perenne* | This study |
|  | AG205041 | *L. multiflorum* | [39] |
|  | *Lp*d03_gp08 | *L. perenne* | This study |
|  | AG205054 | *L. multiflorum* | [39] |
|  | AG205051 | *L. multiflorum* | [39] |
|  | *Lp*HvESTClone2.1 | *L. perenne* | This study |
|  | AG205017 | *L. multiflorum* | [39] |
|  | AG205068 | *L. multiflorum* | [39] |
|  | AG205021 | *L. multiflorum* | [39] |
|  | AG205053 | *L. multiflorum* | [39] |
| C | RGC2BLettuce | *Lactuca sativa* | [38] |
|  | RPS2Arabidopsis | *A. thaliana* | [38] |
|  | AG205027 | *L. multiflorum* | [39] |
|  | *Lp*ESTe11_14rg.2 | *L. perenne* | This study |
|  | *Lp*ESTe11_14rg.1 | *L. perenne* | This study |
|  | *Lp*HvESTClone4.2 | *L. perenne* | This study |
|  | AG205047 | *L. multiflorum* | [39] |
|  | *Lp*NBS-LRR2 | *L. perenne* | This study |
|  | *Lp*NBS-LRR1 | *L. perenne* | This study |
|  | *Lp*HvESTClone2.2 | *L. perenne* | This study |
|  | *Lp*HvESTClone2.3 | *L. perenne* | This study |
| D | *As*PcaClone2 | *Avena sativa* | [37] |
|  | AG205062 | *L. multiflorum* | [39] |
|  | AG205063 | *L. multiflorum* | [39] |
|  | LGFlax | *Linum usitatissium* | [38] |
|  | MFlax | *Linum usitatissium* | [38] |
| E | AG205066 | *L. multiflorum* | [39] |
|  | AG205026 | *L. multiflorum* | [39] |
|  | AG205028 | *L. multiflorum* | [39] |
|  | AG205034 | *L. multiflorum* | [39] |
|  | AG205069 | *L. multiflorum* | [39] |
|  | PRFTTomato | *Lycopersicon esculentum* | [38] |
|  | *Lp*d07_gp09 | *L. perenne* | [38] |
|  | AG205065 | *L. multiflorum* | [39] |
|  | AG205042 | *L. multiflorum* | [39] |
|  | AG205060 | *L. multiflorum* | [39] |
|  | AG205064 | *L. multiflorum* | [39] |
|  | NBSRPH1Rice | *Oryza sativa* | [38] |
|  | *Lp*NBSC8 | *L. perenne* | This study |
|  | *Lp*NBSC1 | *L. perenne* | This study |
|  | *Lp*RGContig2 | *L. perenne* | This study |
|  | AG205050 | *L. multiflorum* | [39] |
|  | AG205062 | *L. multiflorum* | [39] |
|  | AG205045 | *L. multiflorum* | [39] |
|  | AY923216.1 | *Lolium spp* | [38] |
|  | AY923218.1 | *Lolium spp* | [38] |
|  | AG205061 | *L. multiflorum* | [39] |
|  | AY923221.1 | *Lolium* spp | [38] |
|  | *Lp*NBSC15 | *L. perenne* | This study |
|  | *Lp*a11_gp09 | *L. perenne* | This study |
|  | *Lp*RGContig1 | *L. perenne* | This study |
| F | AG205011 | *L. multiflorum* | [39] |
|  | AG205019 | *L. multiflorum* | [39] |
|  | AG205033 | *L. multiflorum* | [39] |
|  | AG205035 | *L. multiflorum* | [39] |
|  | AG205056 | *L. multiflorum* | [39] |
| G | AG205018 | *L. multiflorum* | [39] |
|  | AG205009 | *L. multiflorum* | [39] |
|  | AY923215.1 | *Lolium* spp | [38] |
|  | AG205037 | *L. multiflorum* | [39] |
|  | *Lp*RG2NBS | *L. perenne* | This study |
|  | *Lp*RG1NBS | *L. perenne* | This study |
|  | AY923221.1 | *Lolium* spp | [38] |
|  | AG205038 | *L. multiflorum* | [39] |
|  | *Lp*d02_gp08 | *L. perenne* | This study |
|  | *Lp*NBSC5 | *L. perenne* | This study |
|  | *Lp*ESTa10_13rg.2 | *L. perenne* | This study |
|  | AG205023 | *L. multiflorum* | [39] |
|  | AG205057 | *L. multiflorum* | [39] |
|  | AG205025 | *L. multiflorum* | [39] |
|  | AG205039 | *L. multiflorum* | [39] |
|  | AY923219.1 | *Lolium* spp | [38] |
|  | NBSHordeum | *H. vulgare* | [38] |
|  | AG205058 | *L. multiflorum* | [39] |
|  | AG205020 | *L. multiflorum* | [39] |
|  | AG205036 | *L. multiflorum* | [39] |
| H | AG205055 | *L. multiflorum* | [39] |
|  | *Lp*PcaClone3.1 | *L. perenne* | This study |
|  | AY923223.1 | *Lolium* spp | [38] |
|  | AY923223.1 | *Lolium* spp | [38] |
|  | AY923223.1 | *Lolium* spp | [38] |
|  | DQ010112.1 | *Lolium* spp | [38] |
|  | AY923223.1 | *Lolium* spp | [38] |
|  | AY923223.1 | *Lolium* spp | [38] |
|  | AY923223.1 | *Lolium* spp | [38] |
