# Supplementary material for: Molecular characterisation and genetic mapping of candidate genes for qualitative disease resistance in perennial ryegrass (Lolium perenne L.)
Source: BMC Plant Biol. 2009 May 19;9:62. doi: 10.1186/1471-2229-9-62 (PMC2694799; doi:10.1186/1471-2229-9-62)
Supplement: Additional File 11 — Summary information for LAP and SNuPe primers used for predicted R gene SNP validation. Information on segregation structure, parental polymorphism, SNP variant and successful genetic map assignment is included. All LAP PCRs and SNuPe reactions were designed for operating annealing temperatures of 55°C and 50°C, respectively. [file 1471-2229-9-62-S11.doc]

**Additional File 11**

| **Gene name** | **SNuPe primer**  **designation (numerical identifier, nucleotide coordinate, SNP variant)** | **Forward LAP sequence (5’-3’)** | **Reverse LAP sequence (5’-3’)** | **SNuPe primer sequence (5’-3’)** | **Validation**  **in subset of 10 (F_1_[NA_6_ x AU_6_]) F_1_s** |
| --- | --- | --- | --- | --- | --- |
| *Lp*LrK10 | xlprg1-369ct | GCGTTCAGCTCACAAAGGGATAGAG | GCCACCGTCAACAAAAGAGCAACA | GCAAAATGTAGAGTAAATA | NA_6_ |
| *Lp*PcaClone4.1 | xlprg8-271ct | CCTGAAAAGTGGGATACATATC | AGCTGCCTATTGCTTTTG | CAGCCAGTGGAGGCCTTTCAA | NA_6_ |
| *Lp*RGContig1 | xlprg12-635ct | GGAAGACAACGCTGGCTAAA | CACGCATGACAGACCTTTTG | TTGTATGATGGAAAAGCT | NA_6_ |
| *Lp*RGContig2 | xlprg13-341ct  xlprg13-380ag | CAGTGAGTGCAACCTTCCAA | GCAAGGGTAGACCACCACAT | ATGGAAGAATGGGATG  CCTTTCAGTGGTTCCTCTGTG | AU_6_  AB X AB |
| *Lp*RG1NBS | xlprg15-118ag  xlprg15-277gt  xlprg15-363at | CCTTTTCATCCAGACCATTCA | GGGGAAAACGACACTTGCTA | ACTGTTAGCCCCATTGAG  TACTCTTGTCTATCTTTACA  TTGCAAATCGAAATGATCTG | NA_6_  NA_6_  NA_6_ |
| *Lp*RG2NBS | xlprg16-256gt  xlprg16-104gc | TGGCCCTCGTAGTTCTCATT | TTGCAAATGTGGGTCTGTGT | TACTCTTGTCTATCTTTACA  GGTCTGGATGAAAAGGA | NA_6_  NA_6_ |
| *Lp*ESTa03_10rg.1 | xlprg23-337ct  xlprg23-177ag | ACCAAGGAGTTCATACAGTC | ACCAGTGCTATCCAAGTGTTGATA | GGTGGTTATGGTCCTAGTTG  TGGGGGCATAGGAAAGACA | AU_6_  AU_6_ |
| *Lp*ESTa08_14rg | xlprg24-303ct  xlprg24-345ct  xlprg24-460at | TTAGGGCATCGTAGAACAGCTGAA | CTTTAGGAATAGACGCTC | ATTTGGGTATGCGTCTC  AACCTGGAAATGGCTCTTCAT  ACGGGAGGGATAGCCAAAAAA | NA_6_  NA_6_  NA_6_ |
| *Lp*ESTa10_13rg | xlprg25-608ag | ACCAAGGAGTTCATACAGT | CATCCTCACATAACTTTCCCCTCTA | TCGTCCCACACATCGTCAAGA | NA_6_ |
| *Lp*ESTb06_11rg | xlprg26-153ga  xlprg26-298gt  xlprg26-716ct | AAGTTTCCAGTTCTCCGAGTA | GATGACAACCAGAGAAACAGCAA | CAATTAGGAGAACTACA  CCCACCTGACTTTGATCTCTC  AAAAGAGGAAACATTAGAGAT | AU_6_  AU_6_  AU_6_ |
| *Lp*ESTc10_19rg | xlprg27–743ag  xlprg27-912ag | ATTAATACAGAGGGTGGACAAGAT | GTTTTGGGCAGTTTTCTTATC | GAGTACTTGACATAGAAAATGG  GACATGCTCTTGGGAATAACA | AB X AB  AB X AB |
| *Lp*ESTd08_13rg | xlprg28-509ct  xlprg28-340cg  xlrg28-319cg  xlprg28-Ind519ca | CACTTCATCACTTGCATCG | CCGTATGGTTTAGCGTCCTG | CGCAAGCTAAAAGGAACCATT  TGTCCTGCCCTGAAATCGCT  AGCGATTTCAGGGCAGGACA  CATTGGTCGTGTACAGAA | NA_6_  AU_6_  AU_6_  NA_6_ |
| *Lp*ESTe11_14rg.1 | xlprg29-293ct  xlprg29-156cg | AAGGAGGTTTCTGTTGTTG | CATCCTCACATAACTTTCCCCTCT | TTAAAAAGAGCTTGCCTG  CTATTTGAGAGGACACTC | NA_6_  NA_6_ |
| *Lp*ESTe11_14rg.2 | xlprg30-658ct  xlprg30-707ag | AAGGAGGTTTCTGTTGTTG | CATCCTCACATAACTTTCCCCTCTA | TCGGAATAGGCAGATGGTA TGTGTGGAACCTTGTGGTAC | AU_6_  AU_6_ |
| *Lp*ESTe11_14rg.3 | xlprg31-490ct | AAGGAGGTTTCTGTTGTTG | CATCCTCACATAACTTTCCCCTCTA | GGTGGTTTGGGCTCTGTTCATAG | NA_6_ |
| *Lp*ESTf06_19rg.1 | xlprg40-352ct  xlprg40-284ga  xlprg40-31cg | ACTGTTGAGGCAAGCTCA | GGGCCTTCACTCTGGACAATCTGT | AAGACTCATTTTCATGCCT TGCCTCCTACTCTTTCAAA  CTGTCAAAGCACCAAGAT | NA_6_  NA_6_  NA_6_ |
| *Lp*ESTg01_20rg | xlprg42-331ag | AACCAATTACAGCATCATCACCG | GTGCTGAATCTCTCCTACAATGAT | ATCCCACAAACGCAAACAACA | AU_6_ |
| *Lp*ESTg04_17rg.1 | xlprg43-271ct | ATTTCGTCCACACCGCCATGCTATCCTCG | GGTACTTGAGATTTGCCGC | GAGAAACTACCAAGAGGGATTGC | NA_6_ |
| *Lp*ESTg06_13rg | xlprg44-514ag | TAGTACGGGTGGAAGTTGAGGGAA | AAATAGAGGAGAAGAACTC | TGGGGTCAAGGTGTGGGT | NA_6_ |
| *Lp*ESTg10_13rg.1 | xlprg45-86gt  xlprg45-196ct | GGGTTGGTCATTTTATGGGCTG | TAAGAGGAATGAAATGGCTGTACT | GTATCTTGGTAGTTGTTGACA GAGGTTGGAGAGGGGATAG | NA_6_  NA_6_ |
| *Lp*ESTh04_17rg | xlprg47-625gt  xlprg47-143ct | GCGCCTGCTCTTCTCCACCA | AAGTTGTGGGCAGTTCGGCA | ACTGCCCACAACTTCCCG  CGAATGGTTGGTCACCCT | AU_6_  AA X BB |
| *Lp*ESTh05_28rg.1 | xlprg48-200ct  xlprg48-200ga | CACATAAGCAAAGGGTTCCC | TTACCAATGTCTATCCTACCCCTA | ATGGTGATAGATAAGTG  TGCCATCCCACAATCTGTTCG | AU_6_  AU_6_ |
| LPCL_8913 | xlprg49-105ca xlprg49-27rct  xlprg49-550ga | AAAACTTGCTATGAGCACTTC | GTACCCGAAAATCACAAATTC | CCTAAAGCATCTTCGGTA  ACAGTTCCCATTTGGTGAGCCTA  AGCTGAAATCTGGGTAA | NA_6_  NA_6_  AU_6_ |
| *Lp*HvESTClone1.1 | xlprg50-464ca  xlprg50-441ga  xlprg50-719ga | CAGGAGCAAGAACAGAGTGAC | GGTTTGGACTTTTCCGTATC | CACAAGCTCCAAAAAGGAAC CCTTTTTGGAGCTTGTGCC  CAAGTGACGGGGGAAGCA | NA_6_  AU_6_  NA_6_ |
| *Lp*HvESTClone2.1 | xlprg54-688ga  xlprg54-665cg | GCAGAGAGAATGGAGTAGCACAA | CTTGGAGTGTCTTCTTTATTGC | CAGAGCTTGTTAGTGATGAGGA  CAGAGCTTGTTAGTGATGAGGA | NA_6_  NA_6_ |
| *Lp*HvESTClone3.1 | xlprg55-174ag | CAGTGCCGTCTCGAAGTT | CATCCTCTTGCTCCGTACT | TCCTCCCTCACCAAATTATC | NA_6_ |
| *Lp*HvESTClone4.1 | xlprg56-399ag | TCCTTGTAAGAGTAGTC | GAACCTAGTCCTCACCAG | GAAGCAAGTGAGGGACCT | NA_6_ |
| *Lp*AG205017 | xlprg60-216gt  xlprg60-81cg | GATGTTTGGATAGACCTGGCA | GAAGATGCTGCAGAACACGA | TATGATCTCTCTCCCCAAT  ATTAGGTGATCATAGTACTTA | AB X AB  AB X AB |
| *Lp*AG205018 | xlprg61-23ag  xlprg61-265cg | TGAGGACAGAACAAATTGGG | TTGGGTAAAGGCTGCAGAAG | ACTTACCTGTCCAACTGAA  TCTGGTGATGTTATGAA | AU_6_  AU_6_ |
| *Lp*AG205035 | xlprg62-159ag | GGCTGAAGCTTCTCCTCAAG | AGGTGGCATTTGGTCATAGG | TGAACAGCTCCCAACAAT | NA_6_ |
| *Lp*AG205050 | xlprg63-55ga | GGAGCAATGGGCTGCTATAA | CATTTCTGCAGCAGCTTCGT | ATATCCGATGGTAAGTC | NA_6_ |
| *Lp*AG205055 | xlprg64-81at  xlprg64-172ac  xlprg64-265gc  xlprg64-307ac | GAGCAGCCAATCAGAGAACC | AAATGGTCAGGAAGATGCTCA | CAGAAAGTAATAGTTACGAC  CGCATCACCAAACGCACATT  GTTGGCAGGATCACCTTTA  GACAGCCCTCCAATGATTTT | AU_6_  AA X BB  AB X AB  AB X AB |
| *Lp*AG205063 | xlprg65-202gt | ACTGGAGGATGTTGGTGTCC | CCTCCACAACTTTGGGCTAT | TGCTCTGGCTGTTGGTATC | NA_6_ |
