# Supplementary material for: Molecular characterisation and genetic mapping of candidate genes for qualitative disease resistance in perennial ryegrass (Lolium perenne L.)
Source: BMC Plant Biol. 2009 May 19;9:62. doi: 10.1186/1471-2229-9-62 (PMC2694799; doi:10.1186/1471-2229-9-62)
Supplement: Additional File 12 — Comparative chromosomal positions of predicted putative orthologous R genes between perennial ryegrass and barley: Lps-217 (coded as xlprg50-464ca) on p150/112 LG2 compared to Hvs-217 at the bottom of chromosome 2H. qLr represents a QTL for barley leaf rust resistance. [file 1471-2229-9-62-S12.ppt]

## Slide 1
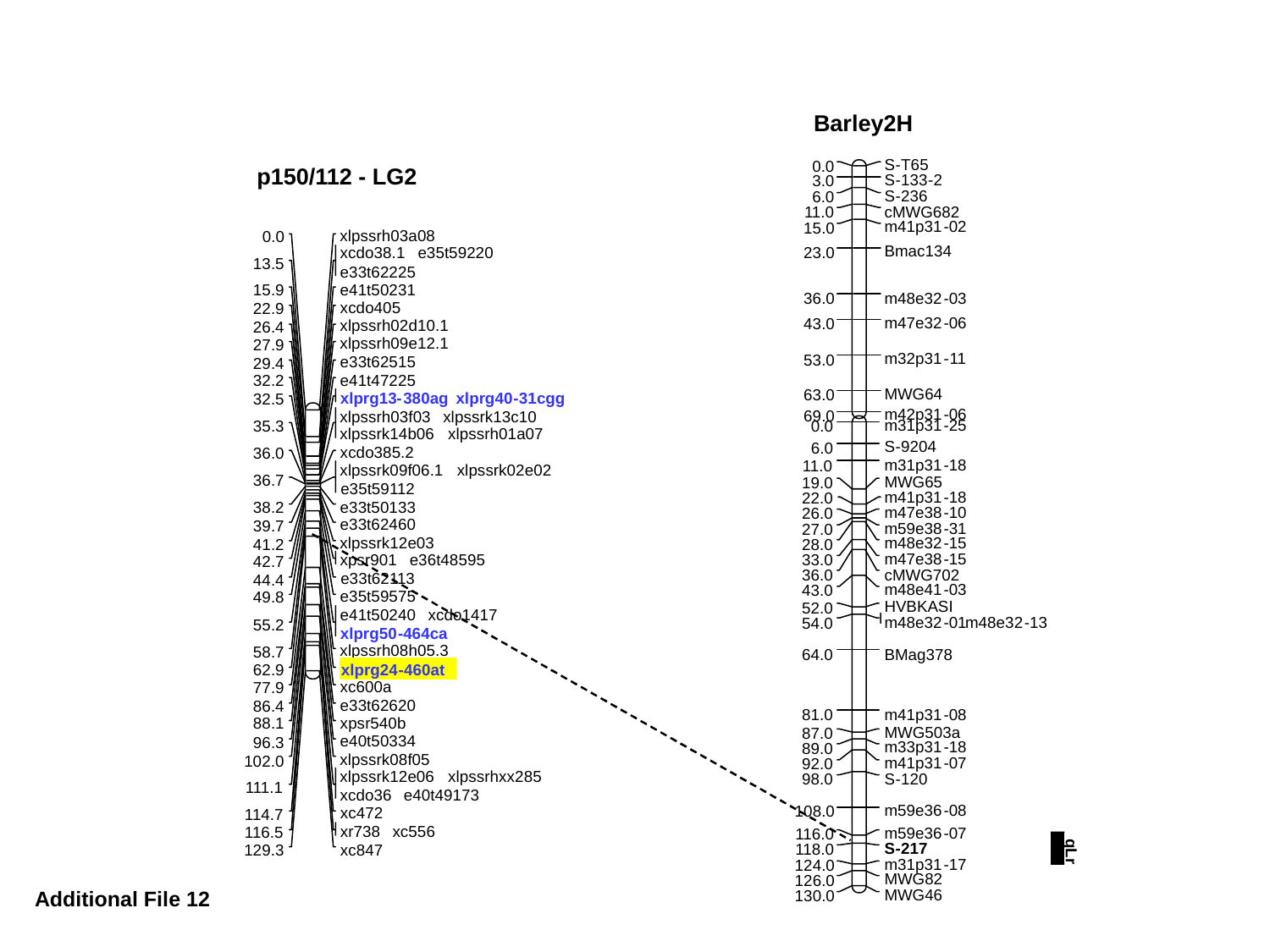

Barley2H
S
-
T65
0.0
S
-
133
-
2
3.0
S
-
236
6.0
11.0
cMWG682
m41p31
-
02
15.0
Bmac134
23.0
36.0
m48e32
-
03
m47e32
-
06
43.0
m32p31
-
11
53.0
MWG64
63.0
m42p31
-
06
69.0
m31p31
-
25
0.0
S
-
9204
6.0
m31p31
-
18
11.0
MWG65
19.0
m41p31
-
18
22.0
m47e38
-
10
26.0
m59e38
-
31
27.0
m48e32
-
15
28.0
m47e38
-
15
33.0
36.0
cMWG702
m48e41
-
03
43.0
HVBKASI
52.0
m48e32
-
01
m48e32
-
13
54.0
64.0
BMag378
81.0
m41p31
-
08
MWG503a
87.0
m33p31
-
18
89.0
m41p31
-
07
92.0
98.0
S
-
120
m59e36
-
08
108.0
m59e36
-
07
116.0
S
-
217
118.0
qLr
m31p31
-
17
124.0
MWG82
126.0
MWG46
130.0
p150/112
-
LG2
xlpssrh03a08
0.0
xcdo38.1
e35t59220
13.5
e33t62225
15.9
e41t50231
xcdo405
22.9
xlpssrh02d10.1
26.4
xlpssrh09e12.1
27.9
e33t62515
29.4
32.2
e41t47225
xlprg13
-
 380ag
xlprg40
-
31cgg
32.5
xlpssrh03f03
xlpssrk13c10
35.3
xlpssrk14b06
xlpssrh01a07
xcdo385.2
36.0
xlpssrk09f06.1
xlpssrk02e02
36.7
e35t59112
38.2
e33t50133
e33t62460
39.7
xlpssrk12e03
41.2
xpsr901
e36t48595
42.7
e33t62113
44.4
e35t59575
49.8
e41t50240
xcdo1417
55.2
xlprg50
-
464ca
xlpssrh08h05.3
58.7
xlprg24
-
460at
62.9
xc600a
77.9
e33t62620
86.4
88.1
xpsr540b
e40t50334
96.3
xlpssrk08f05
102.0
xlpssrk12e06
xlpssrhxx285
111.1
xcdo36
e40t49173
xc472
114.7
xr738
xc556
116.5
129.3
xc847
Fig 46a
Additional File 12
