# Supplementary material for: Molecular characterisation and genetic mapping of candidate genes for qualitative disease resistance in perennial ryegrass (Lolium perenne L.)
Source: BMC Plant Biol. 2009 May 19;9:62. doi: 10.1186/1471-2229-9-62 (PMC2694799; doi:10.1186/1471-2229-9-62)
Supplement: Additional File 13 — Comparative chromosomal positions of predicted putative orthologous R genes between perennial ryegrass and barley: Lps-L8 (coded as xlprg54-688ag) on NA6-LG3 compared to Hvs-L8 at the bottom of chromosome 3H. qMIL represents a major QTL for powdery mildew resistance in barley. [file 1471-2229-9-62-S13.ppt]

## Slide 1
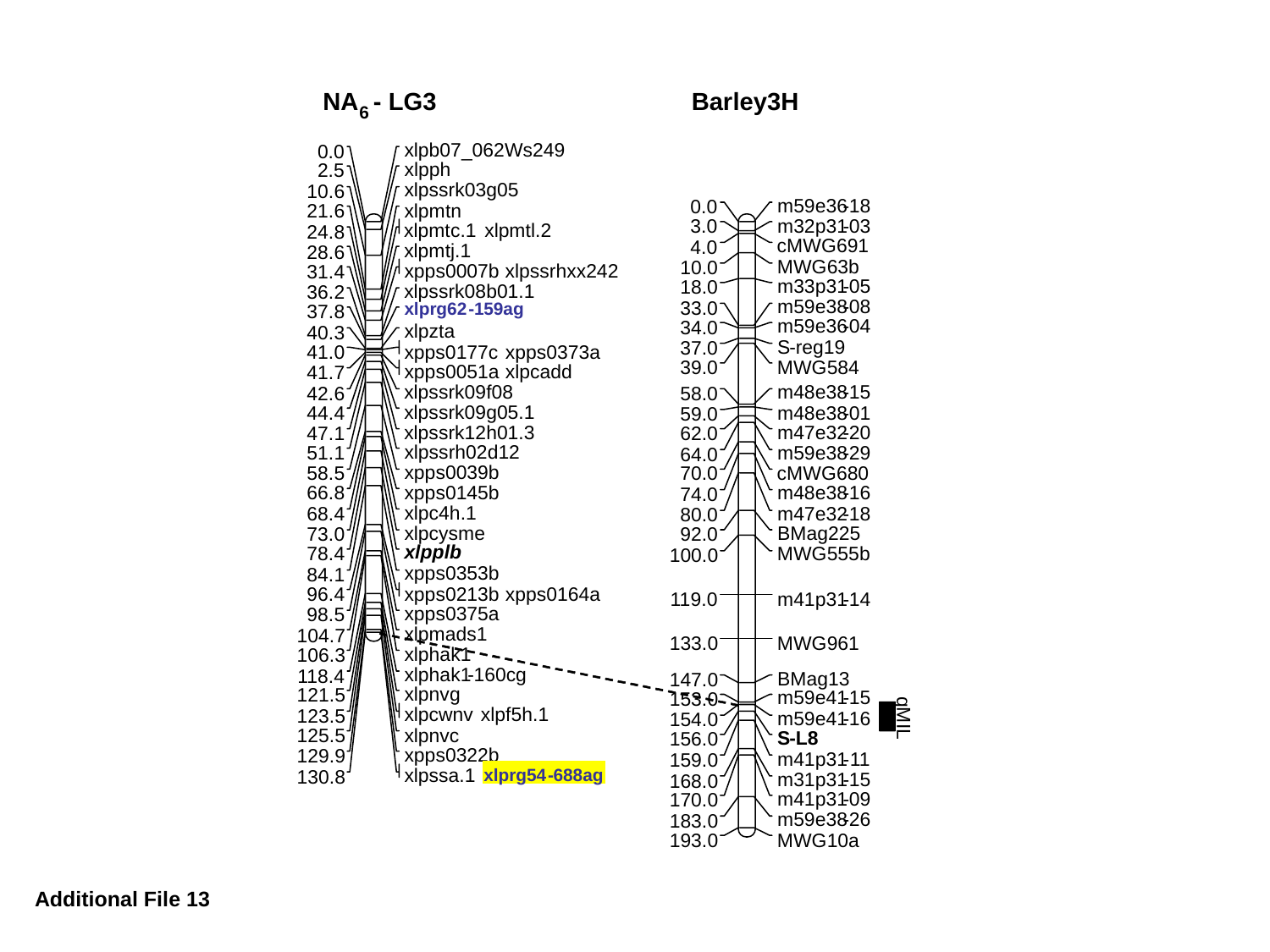

NA
-
LG3
Barley3H
6
xlpb07_062Ws249
0.0
xlpph
2.5
xlpssrk03g05
10.6
m59e36
-
18
0.0
21.6
xlpmtn
3.0
m32p31
-
03
xlpmtc.1
xlpmtl.2
24.8
cMWG691
4.0
xlpmtj.1
28.6
MWG63b
10.0
xpps0007b
xlpssrhxx242
31.4
m33p31
-
05
18.0
xlpssrk08b01.1
36.2
m59e38
-
08
33.0
xlprg62
-
159ag
37.8
m59e36
-
04
34.0
xlpzta
40.3
S
-
reg19
37.0
41.0
xpps0177c
xpps0373a
39.0
MWG584
xpps0051a
xlpcadd
41.7
xlpssrk09f08
m48e38
-
15
42.6
58.0
xlpssrk09g05.1
44.4
m48e38
-
01
59.0
xlpssrk12h01.3
m47e32
-
20
47.1
62.0
xlpssrh02d12
51.1
m59e38
-
29
64.0
xpps0039b
58.5
70.0
cMWG680
66.8
xpps0145b
m48e38
-
16
74.0
xlpc4h.1
68.4
m47e32
-
18
80.0
xlpcysme
BMag225
73.0
92.0
xlpplb
78.4
MWG555b
100.0
xpps0353b
84.1
96.4
xpps0213b
xpps0164a
119.0
m41p31
-
14
xpps0375a
98.5
xlpmads1
104.7
133.0
MWG961
xlphak1
106.3
xlphak1
-
160cg
118.4
BMag13
147.0
xlpnvg
121.5
m59e41
-
15
153.0
xlpcwnv
xlpf5h.1
123.5
m59e41
-
16
qMIL
154.0
125.5
xlpnvc
S
-
L8
156.0
xpps0322b
129.9
m41p31
-
11
159.0
xlpssa.1
xlprg54
-
688ag
130.8
m31p31
-
15
168.0
m41p31
-
09
170.0
m59e38
-
26
183.0
193.0
MWG10a
Fig 46b
Additional File 13
